# Multi-omics and machine learning identify ALOX5 as a ferroptosis-associated driver of epithelial barrier dysfunction in chronic rhinosinusitis with nasal polyps^☆^

**DOI:** 10.1016/j.waojou.2026.101456

**Published:** 2026-09-07

**Authors:** Ziheng Huang, Huiqin Zhou, Li Wang, Yuanyuan Yang, Xiaoqiang Chen, Chengxun Li, Hongmeng Yu, Desheng Wang

**Affiliations:** a Department of Otolaryngology, Fujian Medical University Union Hospital, Fuzhou, China; b Department of Otolaryngology, Research Units of New Technologies of Endoscopic Surgery in Skull Base Tumor (2018RU003), Peking Union Medical College Hospital, Chinese Academy of Medical Sciences and Peking Union Medical College, Beijing, 100730, China; c ENT Institute and Department of Otorhinolaryngology, Eye & ENT Hospital, Fudan University, Shanghai, 200031, China

**Keywords:** Machine learning, Endoplasmic reticulum stress, Chronic rhinosinusitis, Epithelial cells, Ferroptosis

## Abstract

Chronic rhinosinusitis with nasal polyps (CRSwNP) is a common chronic inflammatory disease of the upper airway, and its underlying pathogenesis remains incompletely understood. In this study, endoplasmic reticulum stress (ERS)-related gene sets were employed as the analytical starting point to systematically screen and identify core genes closely associated with CRSwNP. Through the integration of weighted gene co-expression network analysis (WGCNA), differential expression analysis, and multiple machine learning algorithms, 4 hub genes (ALOX5, HMOX1, PXDN, and CARD9) were identified, all of which exhibited favorable diagnostic performance for CRSwNP (AUC = 0.863–0.962, *P* < 0.05). RT-qPCR, Western blotting, and immunohistochemistry were performed to validate the aberrant expression of these hub genes in CRSwNP tissues. Notably, the expression level of ALOX5 was significantly positively correlated with the clinical severity of CRSwNP (*R* = 0.40–0.41, *P* < 0.01). Single-cell transcriptomic analysis further revealed that ferroptosis-related signatures were significantly enriched in nasal epithelial cells from patients with CRSwNP. Immunofluorescence staining and primary nasal epithelial cell experiments indicated that ALOX5 expression was negatively correlated with epithelial tight junction proteins and that targeting ALOX5 effectively modulated the ferroptosis process. Collectively, this study identified hub genes with potential diagnostic value and functional relevance in CRSwNP and elucidated the potential involvement of ALOX5-mediated epithelial ferroptosis in disease progression, thereby providing novel insights into the precise diagnosis and targeted therapy of CRSwNP.

## Introduction

Chronic rhinosinusitis with nasal polyps (CRSwNP) is a highly heterogeneous chronic inflammatory disease of the upper airway, characterized by persistent mucosal inflammation, aberrant tissue remodeling, and a high recurrence rate, for which current therapeutic options remain limited in efficacy.^1^^,^^2^ Epidemiological studies have demonstrated that the global prevalence of chronic rhinosinusitis (CRS) has increased significantly in recent years.^3^ Notably, compared with chronic rhinosinusitis without nasal polyps (CRSsNP), CRSwNP is generally associated with more severe clinical manifestations, poorer long-term outcomes, and a higher risk of recurrence, underscoring its substantial clinical and research significance.^4^, ^5^, ^6^

The endoplasmic reticulum (ER) is a central organelle responsible for maintaining cellular homeostasis through the regulation of protein folding, lipid metabolism, and calcium balance.^7^^,^^8^ Under pathological conditions, disruption of ER homeostasis can induce endoplasmic reticulum stress (ERS), thereby activating the unfolded protein response (UPR) to restore cellular equilibrium.^9^^,^^10^ Emerging evidence suggests that ERS and ferroptosis are closely interconnected biological processes rather than independent events. Persistent ER stress may promote ferroptosis through activation of UPR pathways, particularly the PERK/eIF2α/ATF4 signaling axis, which regulates oxidative stress responses and ferroptosis-related molecules such as SLC7A11.^11^^,^^12^ Furthermore, ERS-induced disturbances in lipid metabolism and redox homeostasis may further increase cellular susceptibility to lipid peroxidation and ferroptotic damage.^13^^,^^14^ Conversely, excessive lipid peroxidation during ferroptosis may exacerbate ER dysfunction, suggesting the existence of bidirectional crosstalk between these processes.^15^ Previous studies have indicated that ERS and its associated signaling pathways are closely associated with epithelial injury, immune cell activation, and amplified inflammatory responses in multiple respiratory inflammatory diseases, including asthma, chronic obstructive pulmonary disease, and allergic rhinitis.^16^, ^17^, ^18^ However, systematic characterization of ERS-related molecular features in CRSwNP remains limited.

Nasal epithelial cells constitute the first line of defense in the upper airway and play a pivotal role in maintaining mucosal homeostasis and regulating local immune responses.^19^^,^^20^ Emerging evidence has suggested that under conditions of persistent inflammation and cellular stress, metabolic dysregulation and oxidative stress within epithelial cells may induce ferroptosis, leading to disruption of epithelial barrier integrity and amplification of local inflammatory responses.^21^, ^22^, ^23^ Nevertheless, whether ERS-related genes participate in epithelial ferroptosis in CRSwNP and the underlying regulatory mechanisms have yet to be elucidated.

Therefore, given that epithelial barrier dysfunction is a central pathological feature of CRSwNP, nasal epithelial cells were selected as the primary focus for investigating ferroptosis-related molecular alterations in a cell type-specific context.

Previous studies have primarily focused on ferroptosis-related features or diagnostic models in CRSwNP, with most analyses remaining at the whole-tissue level and limited understanding of cell type-specific functional relevance. Therefore, in this study, ERS-related gene sets were used as the analytical starting point. Transcriptomic analysis was applied to identify differentially expressed genes, machine learning algorithms were used for feature selection and prioritization, and single-cell RNA sequencing data were used to delineate cell type–specific expression patterns in nasal tissues. Finally, by integrating clinical phenotype scoring, ALOX5 was identified as the key gene most strongly associated with nasal polyp severity. Based on the ferroptosis-related signals indicated by the above analyses, it was hypothesized that ALOX5 may be involved in ferroptosis-related epithelial cell injury.

This study provides new insights into mucosal barrier dysfunction and chronic inflammation in CRSwNP and may serve as a theoretical foundation for precision therapeutic strategies.

## Materials and methods

### Gene expression profiles

The datasets GSE136825,^24^ GSE72713,^25^ and GSE179265^26^ were obtained from the GEO database. A total of 42 nasal polyp tissue samples and 28 control samples were selected from GSE136825, and 6 nasal polyp tissue samples and 3 control samples were selected from GSE72713. These 2 datasets were combined to create the discovery set. Additionally, 17 nasal polyp tissue samples and 7 control samples from GSE179265 were used for validation.

### Clinical data collection

All clinical specimens used in this study were obtained from the Eye & ENT Hospital of Fudan University (Shanghai, China). According to the diagnostic criteria outlined in the European Position Paper on Rhinosinusitis and Nasal Polyps 2020 (EPOS 2020), nasal polyp (NP) tissues were collected from the middle meatus of patients with chronic rhinosinusitis with nasal polyps (CRSwNP) undergoing endoscopic sinus surgery. The exclusion criteria were as follows: (1) diagnosis of fungal rhinosinusitis, inverted papilloma, antrochoanal polyps, granulomatosis with polyangiitis, primary ciliary dyskinesia, or cystic fibrosis; (2) age <18 years; (3) acute upper respiratory tract infection within the previous 4 weeks; and (4) aspirin intolerance. Normal control specimens were obtained from the middle or inferior turbinates of patients undergoing septoplasty or nasal fracture surgery. These control participants were confirmed to have no evidence of rhinosinusitis or allergic rhinitis by sinus computed tomography and allergen screening. Due to ethical and practical constraints in obtaining healthy sinonasal tissues from anatomical sites equivalent to nasal polyps, control tissues were collected from turbinate regions, which may introduce potential anatomical and spatial heterogeneity. Written informed consent was obtained from all participants or their legal guardians, and the study was approved by the Ethics Committee of the Eye & ENT Hospital of Fudan University (No. 2025022). In total, 15 control subjects and 30 patients with CRSwNP were enrolled in this study. The clinical characteristics of the participants and detailed sample information are summarized in Supplementary Table S1.

### Identification of differentially expressed genes

In the training set, differential gene expression between the normal control and CRSwNP groups was analyzed using the R package “limma”. Prior to downstream analyses, batch effects between the GSE136825 and GSE72713 datasets were corrected using the removeBatchEffect function from the “limma” package. Differentially expressed genes (DEGs) were identified using the criteria of an adjusted *P* value (adj P) < 0.05 and |log2 fold change (FC)| > 0.5. The results of the differential expression analysis were visualized using the R packages “ggplot2” and “pheatmap” to generate volcano and heatmaps, thereby providing an intuitive representation of significant gene expression changes.

### Weighted gene Co-expression network analysis (WGCNA)

WGCNA was performed on the training set using the R package “WGCNA” to identify gene modules significantly associated with CRSwNP. First, samples were clustered to detect and remove potential outliers. Subsequently, a weighted co-expression network was constructed by selecting an optimal soft-threshold, followed by the generation of a topological overlap matrix (TOM). Hierarchical clustering was performed based on the TOM-based dissimilarity matrix to partition genes into distinct modules. Next, Pearson correlation analysis was conducted to assess the relationships between each module and the CRSwNP phenotype, thereby identifying modules significantly positively correlated with the disease. Finally, the module exhibiting the highest positive correlation with CRSwNP and its constituent genes were selected for subsequent functional analyses and mechanistic studies.

### Protein-protein interaction (PPI) network analysis

PPI networks were constructed using the STRING database and visualized in Cytoscape (v3.10).^27^ Subsequently, modular analysis was performed using the MCODE plugin to identify highly interconnected clusters.^28^ Additionally, network nodes were evaluated using 5 ranking algorithms in the cytoHubba plugin—degree centrality (Degree), maximal clique centrality (MCC), maximum neighborhood component (MNC), density of maximum neighborhood component (DMNC), and edge percolated component (EPC)—with the top 20 potential hub genes selected.^29^

### Machine learning and clinical features analysis

Machine learning approaches were applied to further identify potential hub genes associated with CRSwNP and endoplasmic reticulum stress (ERS). Specifically, LASSO regression was performed using the R package “glmnet”, and significant features were selected based on the minimum λ value.^30^ Subsequently, support vector machine-recursive feature elimination (SVM-RFE) was conducted using the R package “e1071” to identify top-ranked potential biomarkers.^31^ Additionally, a random forest (RF) model was constructed using the R package “randomForest” to assess the importance of feature genes, with genes scoring above 2 selected.^32^ Furthermore, an artificial neural network (ANN)-based predictive model was constructed using the R packages “neuralnet” and “neuralnettools”,^33^ and model performance as well as the robustness of candidate gene prediction were evaluated using receiver operating characteristic (ROC) curves.To further validate clinically relevant hub genes, the correlations between the expression of hub genes and patients' clinical features were analyzed using GraphPad Prism (v10.2.0).

### Single-cell RNA-seq data processing and analysis

Single-cell RNA-seq data from the HRA000772 dataset^34^ were processed and analyzed using the Seurat R package (version 5.1.0). Cells expressing fewer than 300 genes, with fewer than 1000 total RNA counts, or with mitochondrial gene content exceeding 20% were excluded. Data were normalized using the *NormalizeData* function, and the top 2000 highly variable genes were identified with *FindVariableFeatures*. The normalized data were subsequently scaled using *ScaleData*. Principal component analysis (PCA) was performed for dimensionality reduction, followed by clustering and visualization using Uniform Manifold Approximation and Projection (UMAP). Differentially expressed genes were identified using *FindAllMarkers* with thresholds of |log2FC| > 0.5 and adjusted *P* < 0.05. Cell clusters were annotated based on established markers from previous studies,^34^ with detailed annotation strategies provided in Table S5. Gene set variation analysis (GSVA) was applied to evaluate cell death–related pathways in nasal epithelial cells, using gene sets listed in Table S6.^35^ Cell–cell communication networks were inferred using the CellChat R package.^36^

### Construction of the molecular regulatory network

To construct a molecular regulatory network for ALOX5, the HTFtarget database was first used to predict transcription factors (TFs) directly interacting with ALOX5.^37^ Subsequently, mRNA–miRNA interactions of ALOX5 were predicted using online tools such as PITA, microT, and TargetScan, with only those interactions consistently identified by all tools considered reliable. Next, miRNA–lncRNA interactions were screened using the miRNet, starBase, and lncBase v3 databases, retaining only high-confidence interactions validated across multiple databases. Finally, the LncACTdb 3.0 online database was used to predict transcription factors potentially interacting with the selected lncRNAs, thereby completing the construction of an integrated ALOX5–TF–miRNA–lncRNA regulatory network.^37^

### Identification of small molecule drugs

The Connectivity Map (CMap) is a gene expression-based database that reveals potential functional connections among drugs, genes, and diseases by analyzing cellular responses to various perturbations.^38^ In this study, CMap analysis was performed to predict small-molecule compounds that could target subtypes associated with inflammatory responses. Specifically, drug signatures were retrieved from the CMap database (https://clue.io/), and the top 100 upregulated and top 100 downregulated genes showing the most significant expression changes in each subtype were used as input. Each small-molecule compound was assigned a score ranging from −100 to 100, with negative scores indicating the ability of the compound to reverse gene expression patterns, suggesting potential therapeutic effects. Based on the ranking of these scores, the top 5 compounds with the lowest CMap scores were selected as candidate drugs.

### Molecular docking analysis

Molecular docking techniques were employed to investigate the binding affinity between the characteristic gene proteins and small molecules.^39^ Protein structures of the characteristic genes were sourced from the Protein Data Bank (PDB), while molecular structures of small molecules were obtained from PubChem. Ligand and receptor preparations were performed using AutoDockTools, including the addition of hydrogen atoms and identification of the active binding site. Binding modes between the candidate proteins and small molecules were analyzed using AutoDock Vina software.

### Isolation and culture of primary human nasal epithelial cells (pHNECs)

pHNECs were isolated from tissue specimens obtained from both control subjects and patients with CRSwNP. For the control group, nasal samples were collected from the inferior turbinate, whereas polyp tissues were obtained from CRSwNP patients when available. Tissues were digested overnight in DMEM/F12 medium containing penicillin (200 U/mL), streptomycin (200 mg/mL), and 0.2% Dispase II (Beyotime, #ST2339). Following digestion, the tissues were filtered through a 70 μm cell strainer and centrifuged at 500 g for 5 min. The resulting cell pellet was resuspended in PneumaCult™-Ex Medium (Stemcell, #05008) and seeded onto plastic culture dishes. The medium was replaced on the first day after seeding and subsequently every other day until cells reached 70–80% confluence under humidified conditions at 37°C with 5% CO_2_. Cells were detached using 0.05% trypsin-EDTA (Gibco, #25300062), and passages F1 to F3 were used for subsequent experiments.

### Real-time quantitative PCR (RT-qPCR)

Nasal tissue samples from 13 patients with CRSwNP and 20 healthy controls were retrospectively collected for RT-qPCR validation. Total RNA was reverse-transcribed into complementary DNA (cDNA). Quantitative real-time PCR was performed using the LightCycler® 480 system. The thermal cycling conditions consisted of initial denaturation at 95°C for 5 min, followed by 40 cycles. All reactions were performed in technical duplicates. Gene expression levels were quantified using the 2ˆ^−△△Ct^ method, with GAPDH used as the internal control for normalization. Primer sequences for target genes are provided in Supplementary Table S2.

### Western blot

Total protein was extracted from tissues and cells using RIPA lysis buffer (Beyotime, #P0013B) supplemented with protease inhibitor (MCE, HY-K0010) and phosphatase inhibitor (MCE, HY-K0021). Protein concentrations were determined using a BCA assay (Absin, #abs9232). The lysates were mixed with SDS loading buffer (Beyotime, #P0015L) and denatured at 100°C for 10 min. Protein extracts were loaded onto 10% SDS–PAGE gels, separated by electrophoresis, and transferred onto PVDF membranes (Millipore, USA). The membranes were blocked with a protein-free rapid blocking buffer (Epizyme, #PS108P) at room temperature for 30 min, followed by incubation with primary antibodies diluted in antibody dilution buffer (Epizyme, #PS119L) at 4°C overnight. After washing with TBST, the membranes were incubated with HRP-conjugated secondary antibodies at room temperature for 1 h. Protein signals were detected using an enhanced chemiluminescence reagent (APExBIO, #K1233), visualized using a Bio-Rad® ChemiDoc Touch Imaging System, and quantified with Fiji/ImageJ software (NIH, USA). Primary antibodies used for immunoblotting included anti-ALOX5 (Proteintech, #10021-1-Ig, 1:1000), anti-ACSL4 (Proteintech, #22401-1-AP, 1:30,000), anti-GPX4 (Proteintech, #67763-1-Ig, 1:2000), anti-SLC7A11 (Proteintech, #26864-1-AP, 1:2000), and anti-α-tubulin (Proteintech, #66031-1-Ig, 1:30,000). Secondary antibodies included HRP-conjugated goat anti-mouse IgG (Proteintech, #SA00001-1, 1:20,000) and HRP-conjugated goat anti-rabbit IgG (Proteintech, #SA00001-2, 1:20,000).

### Immunohistochemistry

Collected nasal tissue samples were fixed in 4% paraformaldehyde, dehydrated, embedded in paraffin, and sectioned at 5 μm thickness. Tissue sections were first baked at 65°C for 2 h, followed by deparaffinization, rehydration, and blocking of endogenous peroxidase activity, and then incubated with goat serum to block non-specific binding sites.Sections were deparaffinized, rehydrated, and subjected to endogenous peroxidase blocking, followed by blocking of nonspecific binding sites with goat serum. The sections were then incubated overnight at 4°C with anti-ALOX5 (Proteintech, #10021-1-Ig, 1:1000). After washing with PBS, sections were incubated at room temperature with HRP-conjugated secondary antibodies for 1 h, followed by development with DAB substrate for 1 min. After color development, the sections were dehydrated through graded ethanol, cleared in xylene, and mounted with neutral resin. All sections were imaged under a microscope and analyzed using Fiji/ImageJ software (NIH, USA).

### Immunofluorescence

Paraffin-embedded nasal tissue sections were first baked at 65°C for 2 h, followed by deparaffinization and rehydration. Antigen retrieval was then performed by heating the sections in 0.01 M citrate buffer (pH 6.0) at 100°C for 10 min, followed by cooling to room temperature. The sections were subsequently blocked with 5% BSA at room temperature for 1 h. Sections were incubated overnight at 4°C with primary antibodies against ALOX5 (Proteintech, #10021-1-Ig, 1:100), ZO-1 (Proteintech, #66452-1-Ig, 1:600), and Claudin-1 (Servicebio, #GB15032, 1:500). After washing with PBS, the sections were incubated at room temperature for 1 h with species-matched fluorescent secondary antibodies: Alexa Fluor 488 donkey anti-rabbit (#A21206, 1:300) and Alexa Fluor 594 donkey anti-mouse (#A21209, 1:300). All samples were counterstained with DAPI (APExBIO, #C3362) and imaged using a confocal microscope (Leica). Quantitative fluorescence analysis was performed using Fiji/ImageJ (NIH, USA) employing standardized intensity and colocalization measurement methods.

### Malondialdehyde (MDA) assay

MDA levels were measured using the Lipid Peroxidation MDA Assay Kit (Beyotime, #S0131S). Cells were lysed with RIPA buffer (Beyotime, #P0013B) and centrifuged at 12,000×*g* for 10 min to collect the supernatant. The supernatants were mixed with MDA working solution, heated at 100°C for 15 min, and then cooled. After centrifugation, the supernatants were transferred to a 96-well plate, and absorbance was measured at 532 nm using a microplate reader. MDA concentrations were calculated based on a standard curve.

### Assessment for ROS and Fe^2+^ level

Intracellular reactive oxygen species (ROS) and ferrous ions (Fe^2+^) levels were measured using a ferroptosis fluorescence detection kit containing CM-H2DCFDA and RhoNox-6 probes (Beyotime, #C1190S), according to the manufacturer’s instructions. Briefly, pHNECs were incubated in serum-free medium containing CM-H2DCFDA and RhoNox-6 working solutions at 37°C for 30 min in the dark. After incubation, the staining solution was removed, and Assay Buffer was added for fluorescence imaging. Green fluorescence generated from oxidized CM-DCF indicated intracellular ROS levels, whereas red fluorescence from RhoNox-6 was used to detect intracellular Fe^2+^ accumulation. All fluorescence images were acquired using a Leica confocal microscope, and quantitative image analysis was performed using Fiji/ImageJ (NIH, USA).

### LDH release assay

LDH release was measured using the LDH Cytotoxicity Assay Kit (Beyotime, #C0018S). Briefly, 100 μL of culture supernatant was collected from each well of a 96-well plate and mixed with 100 μL of LDH reaction mixture. The mixture was incubated at room temperature in the dark for 30 min. Subsequently, 20 μL of stop solution was added to each well and mixed thoroughly. Absorbance was measured at 450 nm (signal) and 450 nm (reference) using a microplate reader.

### Cell viability assay

pHNECs were cultured in 96-well plates. Cell viability was assessed using the CCK-8 assay kit (Beyotime, #C0037). CCK-8 solution (10 μL per 100 μL culture medium) was added to each well, followed by incubation at 37°C for 1.5 h. Absorbance at 450 nm was subsequently measured at the same time each day using a microplate reader.

### Statistical analysis

Statistical analyses were conducted using R software (version 4.2.2) and GraphPad Prism 8.0.2 (GraphPad Software Inc., San Diego, CA, USA). Comparisons between 2 groups were performed using a *t*-test, while multiple comparisons were conducted using one-way analysis of variance (ANOVA). A *P*-value of <0.05 was considered statistically significant.

## Result

### Identification of CRSwNP modules

**Fig. 1:**
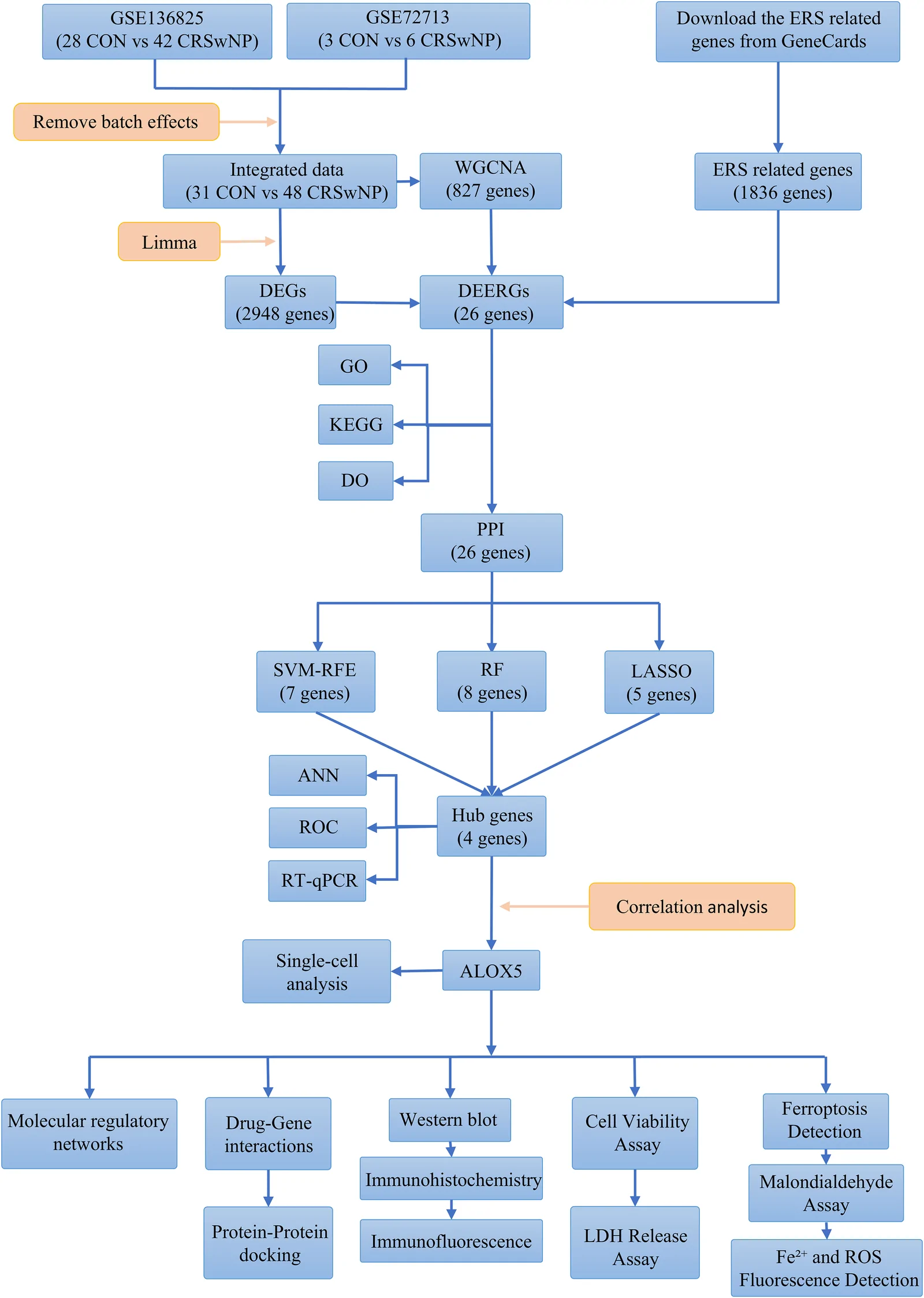
The flowchart illustrating the investigative procedure.

**Fig. 2:**
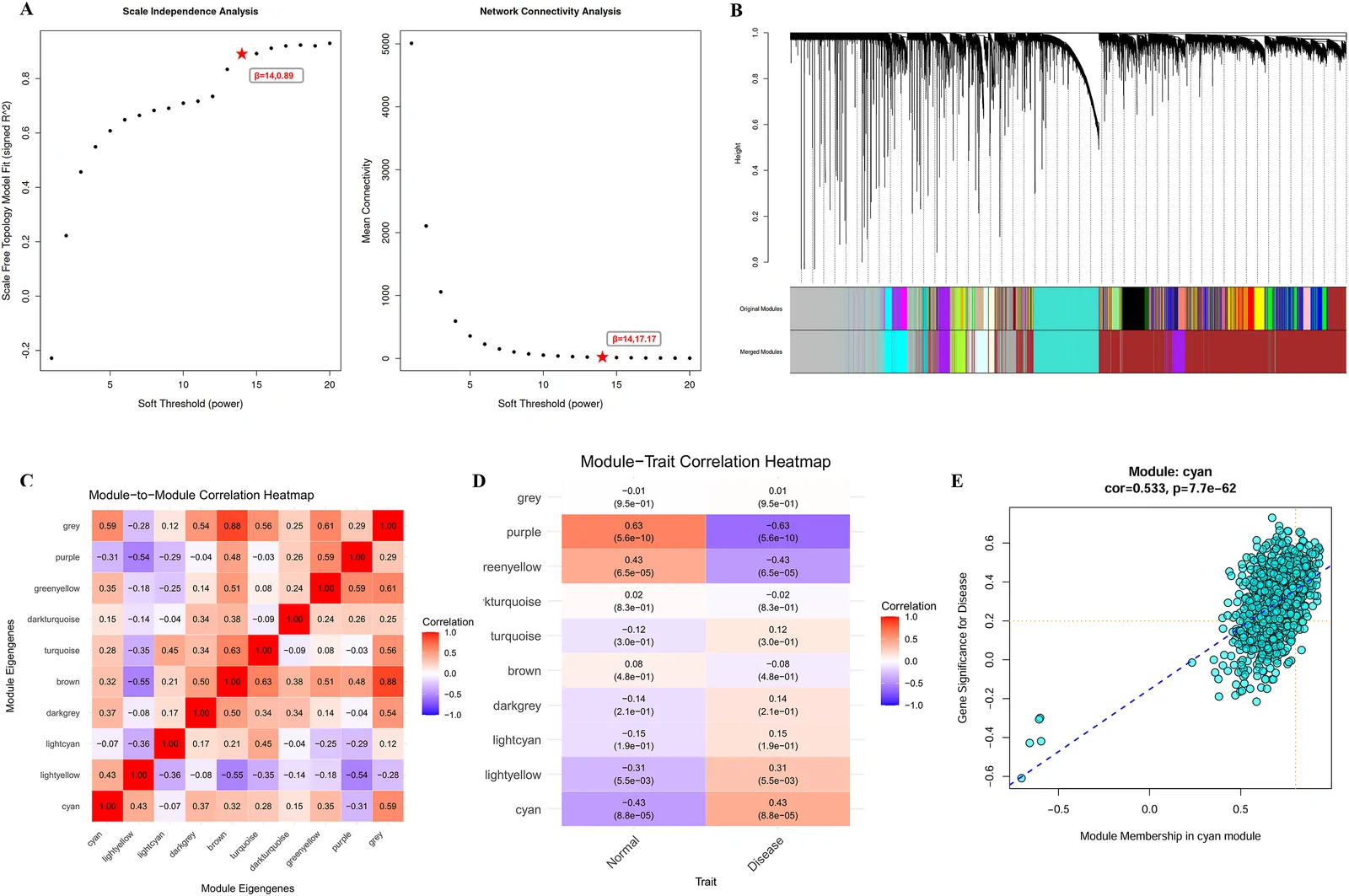
**Identification of CRSwNP-associated gene modules by weighted gene co-expression network analysis**. (A) Selection of the optimal soft-thresholding power for WGCNA: the left panel shows the scale-free topology fit index, and the right panel presents the mean connectivity at different power values.(B) Hierarchical clustering dendrogram of enriched genes showing module assignment based on a dissimilarity measure.(C) Heatmap illustrating the correlations among modules.(D) Heatmap depicting the correlations between module eigengenes and CRSwNP/control samples.(E) Scatter plot showing the correlation between gene membership in the cyan module and gene significance.

The experimental protocol is illustrated in the flowchart (Fig. 1). Weighted Gene Co-expression Network Analysis (WGCNA) was performed to identify gene modules significantly associated with chronic rhinosinusitis with nasal polyps (CRSwNP). The soft-thresholding power (β) was determined to be 14 to construct a scale-free network (Fig. 2A). Using the dynamic tree cut algorithm, genes were clustered into 10 distinct modules, including gray, purple, greenyellow, darkturquoise, turquoise, brown, darkgrey, lightcyan, lightyellow, and cyan (Fig. 2B). Module–trait relationships were subsequently evaluated, indicating that only the cyan module exhibited a significant correlation with CRSwNP (Fig. 2C). Further correlation analysis demonstrated a moderate correlation between the cyan module and gene significance (cor = 0.43, *P* = 8.8 × 10^−5^; Fig. 2D). Moreover, analysis of the relationship between module membership (MM) and gene significance (GS) revealed a strong association across phenotypes (cor = 0.533, *P* = 7.7 × 10^−6^; Fig. 2E). Consequently, the cyan module was identified as the key disease-associated module, comprising 827 genes, which are listed in Supplementary Table S3.

### Identifying the DEGs of CRSwNP

**Fig. 3:**
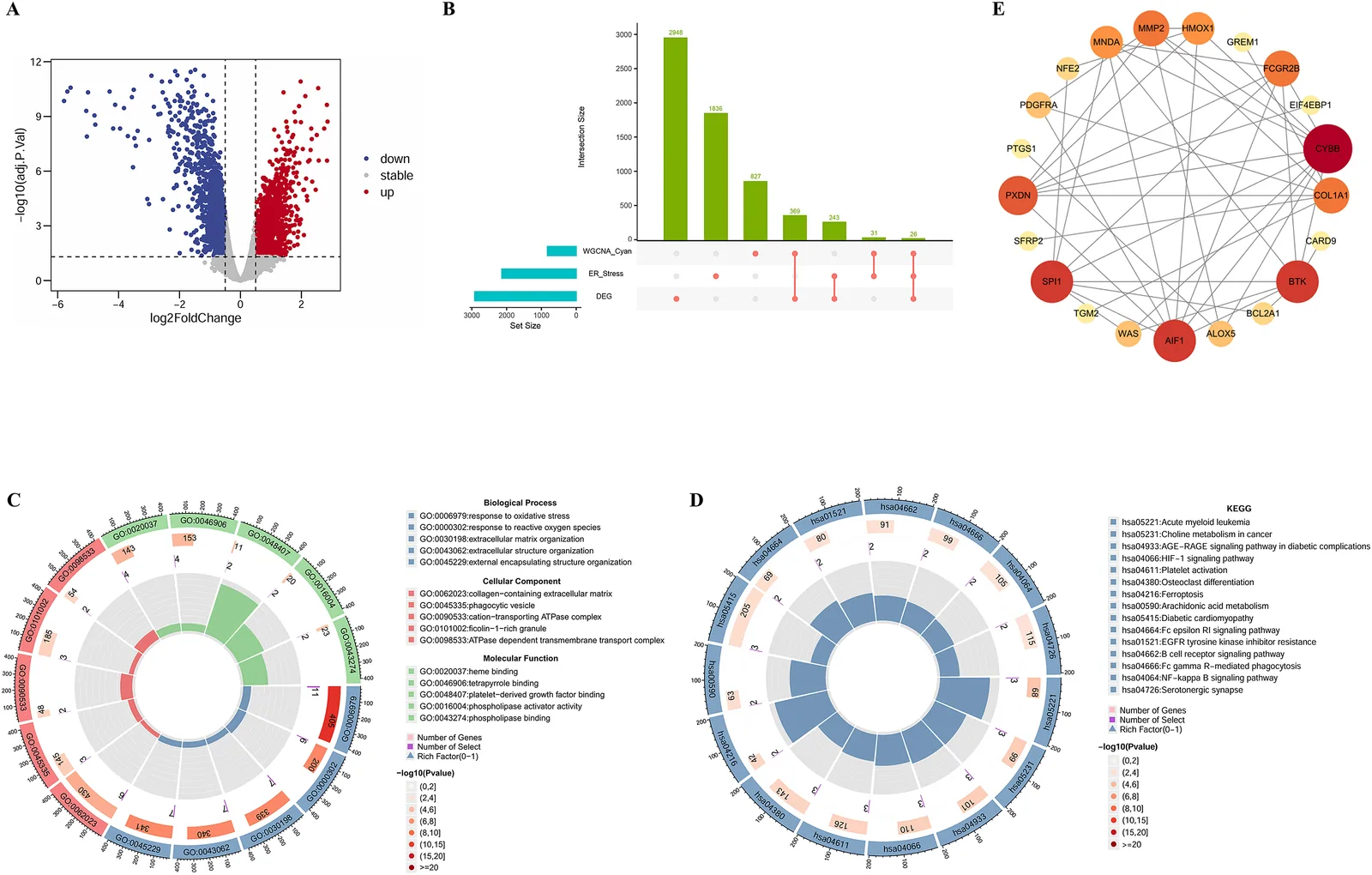
**Identification and functional characterization of endoplasmic reticulum stress (ERS)-related differentially expressed genes in CRSwNP.** (A) Volcano plot displaying the differentially expressed genes (DEGs) between CRSwNP and control groups. (B) UpSet plot demonstrating the intersection among DEGs, ERS-related genes, and the genes most strongly correlated with CRSwNP. (C) Gene Ontology (GO) enrichment analysis of differentially expressed ERS-related genes (DEERGs). (D) Kyoto Encyclopedia of Genes and Genomes (KEGG) pathway enrichment analysis of DEERGs. (E) MCODE analysis: Twenty-one significant genes were identified using the MCODE algorithm.

After batch effect correction, we integrated 48 chronic rhinosinusitis with nasal polyps (CRSwNP) samples and 31 matched control samples for subsequent analysis (Supplementary Fig. 1A). Differential expression analysis revealed 2948 significantly differentially expressed genes (DEGs) with a false discovery rate (FDR) < 0.05, including 1478 upregulated and 1470 downregulated genes. The expression differences of these DEGs between the CRSwNP and control groups were visualized through volcano plots and heatmaps (Fig. 3A and Supplementary Fig. 1B). Additionally, an intersection analysis involving DEGs, genes from the cyan module, and curated endoplasmic reticulum stress (ERS)-related genes identified 26 candidate core genes, which were displayed using an UpSet plot (Fig. 3B).

### Functional enrichment analysis of differentially expressed ERS-related genes (DEERGs) in CRSwNP

To elucidate the potential biological functions of DEERGs in CRSwNP, GO and KEGG enrichment analyses were performed. At the biological process (BP) level, DEERGs were primarily enriched in processes closely related to cellular stress and tissue remodeling, including oxidative stress response, reactive oxygen species (ROS) response, and extracellular matrix organization. These processes represent core features of ERS initiation and sustained activation, which are highly consistent with the chronic inflammation and epithelial injury microenvironment characteristic of CRSwNP. In the cellular component (CC) analysis, DEERGs were significantly enriched in collagen-containing extracellular matrix, suggesting a potential role in aberrant matrix deposition and tissue remodeling during nasal polyp formation (Fig. 3C). Further KEGG pathway analysis revealed significant enrichment of DEERGs in the ferroptosis pathway, as well as multiple signaling pathways closely associated with inflammatory responses and cellular stress, including HIF-1 and NF-κB signaling pathways (Fig. 3D). Ferroptosis, a form of programmed cell death driven by oxidative stress, may play a critical role in epithelial injury and persistent inflammation in CRSwNP. Finally, further disease ontology (DO) analysis demonstrated that DEERGs were primarily associated with various chronic inflammation-related diseases, including coronary artery disease, lower respiratory tract diseases, and nephritis (Supplementary Fig. 1C), suggesting that the underlying molecular mechanisms of CRSwNP may share commonalities with broad stress–inflammation response pathways.

### PPI network and hub gene analysis

**Fig. 4:**
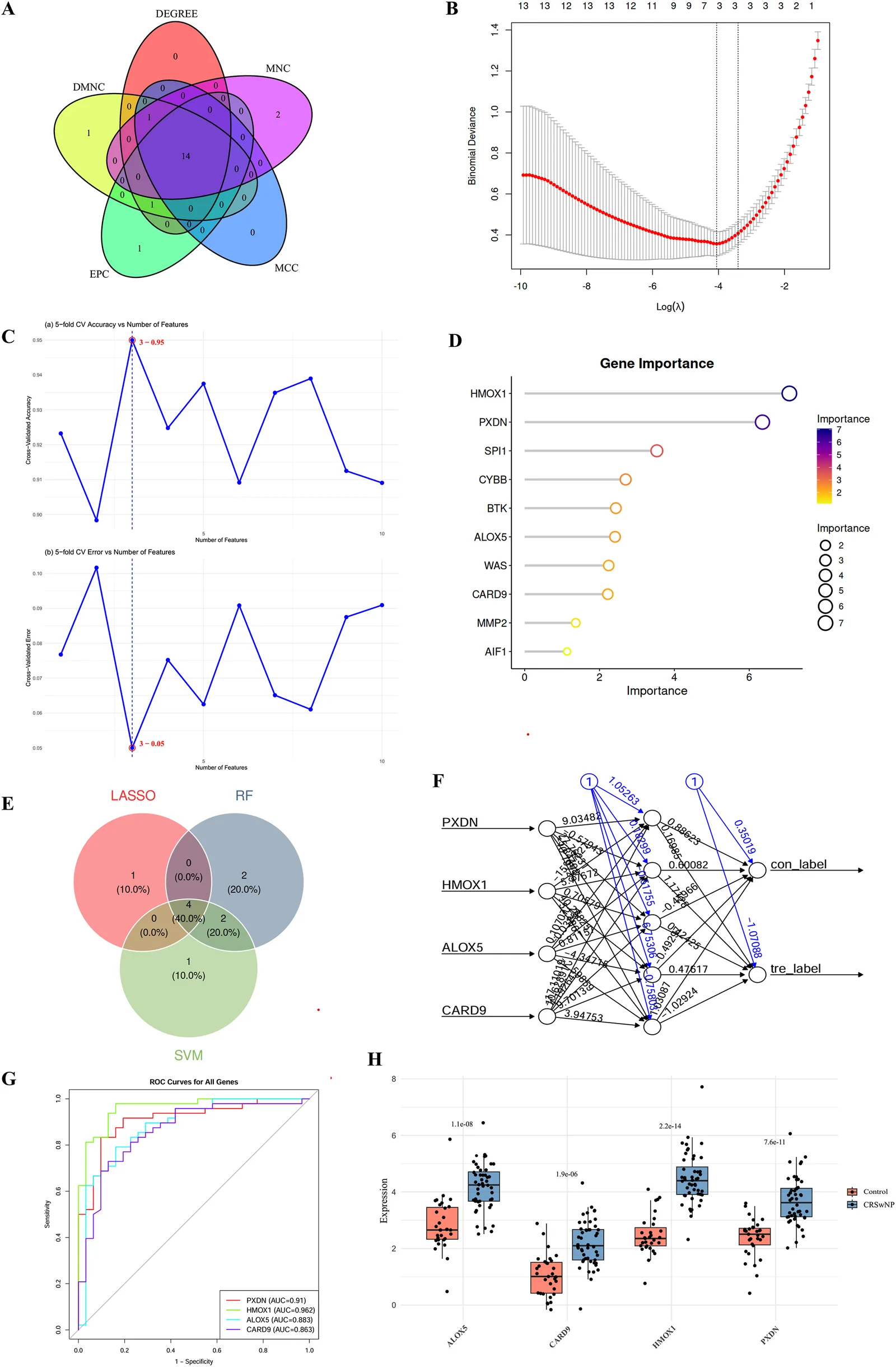
**Machine learning-based screening of hub genes in CRSwNP**. (A) cytoHubba analysis: Fourteen common hub genes were identified using 5 algorithms in the cytoHubba plugin. (B) LASSO regression analysis: Five key genes were selected from the 14 core genes using the LASSO regression machine learning model. (C) Support vector machine (SVM) analysis: Cross-validation accuracy and error curves were generated to determine the optimal number of features in the SVM model. (D) Random forest analysis: Eight key genes were identified from the core genes using the random forest (RF) algorithm. (E) Overlapping analysis: Four overlapping hub genes were identified, including HMOX1, ALOX5, PXDN, and CARD9. (F) ANN model construction: An artificial neural network (ANN) model was constructed based on the identified hub genes. (G) Diagnostic performance: Receiver operating characteristic (ROC) curve analysis was performed to evaluate the performance of the ANN model. (H) Training dataset validation: The expression levels of hub DEERGs were assessed in the training dataset.

To identify the hub genes among the 26 DEERGs, we constructed a protein-protein interaction (PPI) network utilizing the STRING tool. The most densely connected module within this network was identified using the MCODE plugin, which consisted of 21 nodes and 44 edges (Fig. 3E). Additionally, we employed a cross-analysis with 5 ranking algorithms from the cytoHubba plugin, which highlighted 14 common feature genes: CYBB, BTK, SPI1, AIF1, PXDN, MMP2, FCGR2B, COL1A1, HMOX1, MNDA, ALOX5, WAS, PDGFRA, and CARD9 (Fig. 4A). Furthermore, we applied multiple machine learning algorithms. First, LASSO regression analysis identified 5 feature genes at the minimal lambda value (λ.min = 0.019) (Fig. 4B and Supplementary Fig. 2A). Subsequently, the SVM-RFE analysis selected 7 feature genes Fig. 4C), while the random forest (RF) model identified 8 genes with an importance score greater than 2 (Fig. 4D and Supplementary Fig. 2B). The results from these 3 independent methods were integrated using a Venn diagram, which revealed 4 hub genes—HMOX1, ALOX5, PXDN, and CARD9—that were identified as robust biomarkers for the diagnosis of CRSwNP (Fig. 4E).

### Expression of hub DEERGs

An artificial neural network (ANN) diagnostic model was constructed based on gene weight coefficients (Fig. 4F). The diagnostic performance of the model was evaluated using receiver operating characteristic (ROC) curve analysis. The areas under the curve (AUCs) for PXDN, HMOX1, CARD9, and ALOX5 were 0.91, 0.962, 0.883, and 0.863, respectively (Fig. 4G), indicating excellent discriminatory ability in distinguishing CRSwNP from control samples.

To assess the internal performance of the identified hub genes, the batch-effect–corrected merged dataset was used to evaluate their expression patterns. The results confirmed significant differential expression of the hub genes between the CRSwNP and control groups (Fig. 4H). Ridgeline plot analysis demonstrated that the expression distributions of ALOX5 and HMOX1 in CRSwNP samples were generally shifted to the right, indicating consistent upregulation across the population (Supplementary Fig. S2C).

To further assess the robustness and reproducibility of these findings, an independent GEO dataset (GSE179265) was used as an external validation cohort. Consistent results demonstrated that the hub genes were significantly differentially expressed between the CRSwNP and control groups (Supplementary Fig. S2D), supporting the stability of the identified diagnostic signatures.

### ALOX5 expression and clinical relevance

**Fig. 5:**
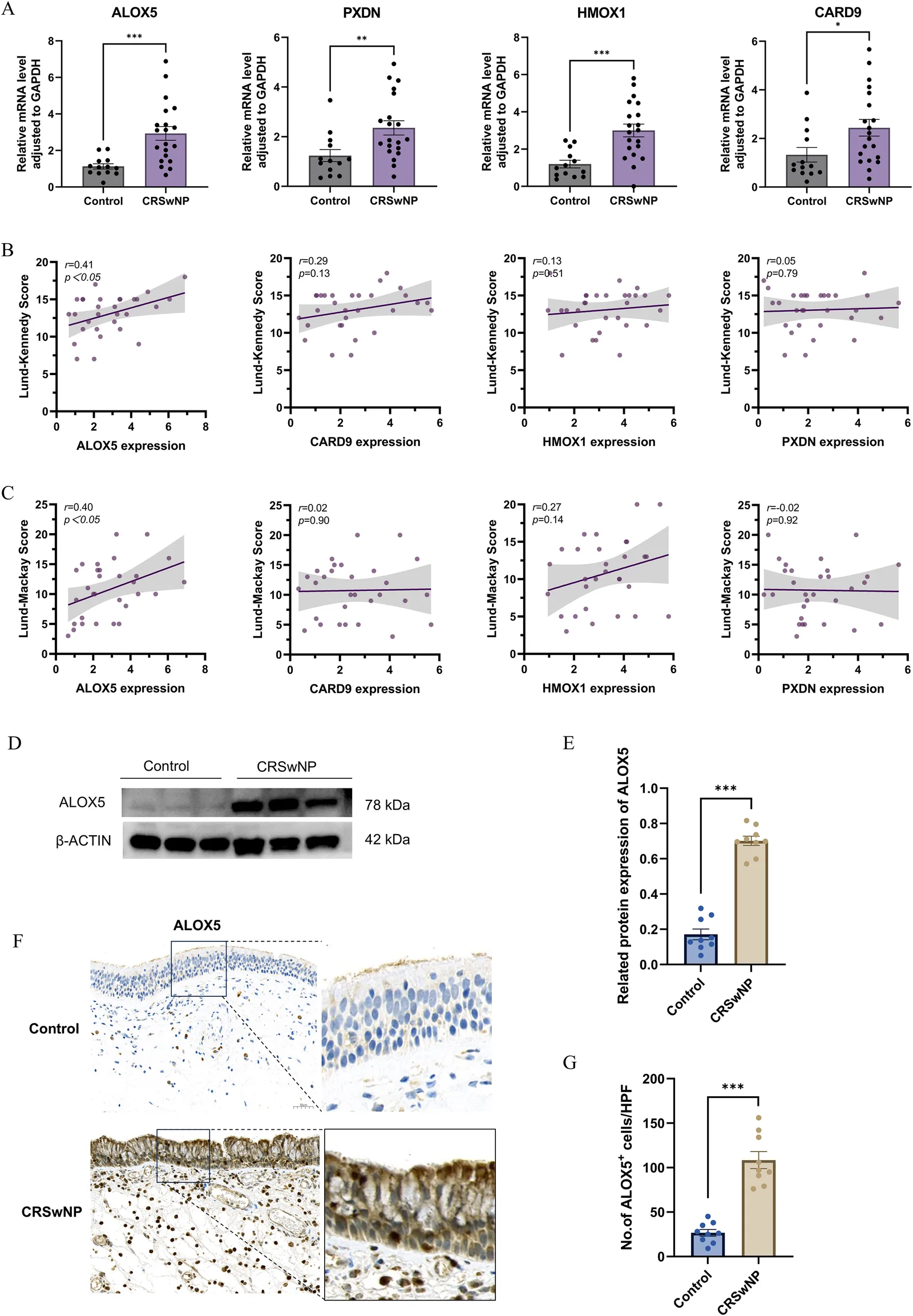
**Expression and clinical correlation of hub genes in CRSwNP**. (A) mRNA expression of hub genes in control and CRSwNP samples. (B–C) The expression of ALOX5 was correlated with clinical parameters, including the Lund–Kennedy and Lund–Mackay scores. Pearson correlation analysis was performed, with r representing the correlation coefficient. (D) Protein expression levels of ALOX5 in control and CRSwNP tissues were detected by Western blotting, with GAPDH used as the loading control. (E) Quantitative analysis of the relative protein expression levels of ALOX5. (F) Immunohistochemical (IHC) staining was performed to assess the localization of ALOX5 expression in control and CRSwNP tissues. Scale bar: 50 μm; magnification: 200×. (G) Quantitative analysis of the number of ALOX5-positive cells. mRNA levels were quantified using reverse transcription quantitative PCR (RT-qPCR), with GAPDH serving as the internal control. Gene expression was normalized using the 2ˆ-ΔΔCT method. Bar graphs represent the mean ± standard deviation (SD). Significance levels: ∗*P* < 0.05; ∗∗*P* < 0.01; ∗∗∗*P* < 0.001; ∗∗∗∗*P* < 0.0001; ns, not significant.

To further elucidate the clinical significance of the identified hub genes in CRSwNP, their expression patterns were validated in clinical tissue samples. RT–qPCR analysis revealed that all hub genes were significantly upregulated in CRSwNP tissues compared with normal control samples (Fig. 5A).

To investigate the relationship between hub gene expression and disease severity, the Lund–Mackay and Lund–Kennedy scores of patients with CRSwNP were assessed (Table S4). Correlation analysis demonstrated that ALOX5 expression was significantly and positively correlated with both the Lund–Kennedy score (*r* = 0.41, *P* < 0.05) and Lund–Mackay score (*r* = 0.40, *P* < 0.05) Fig. 5B and C). These findings indicate that elevated ALOX5 expression is closely associated with increased disease severity, suggesting a potential role in CRSwNP progression and possible clinical predictive value.

Based on these findings, ALOX5 was selected for further in-depth investigation. Western blot analysis demonstrated that ALOX5 protein expression was significantly elevated in CRSwNP tissues compared with control samples (Fig. 5D and E). Immunohistochemical staining further confirmed that ALOX5 expression was markedly increased in both the epithelial layer and lamina propria of CRSwNP tissues (Fig. 5F and G), indicating its potential involvement in local inflammatory responses and tissue remodeling processes.

### Single-cell analysis

**Fig. 6:**
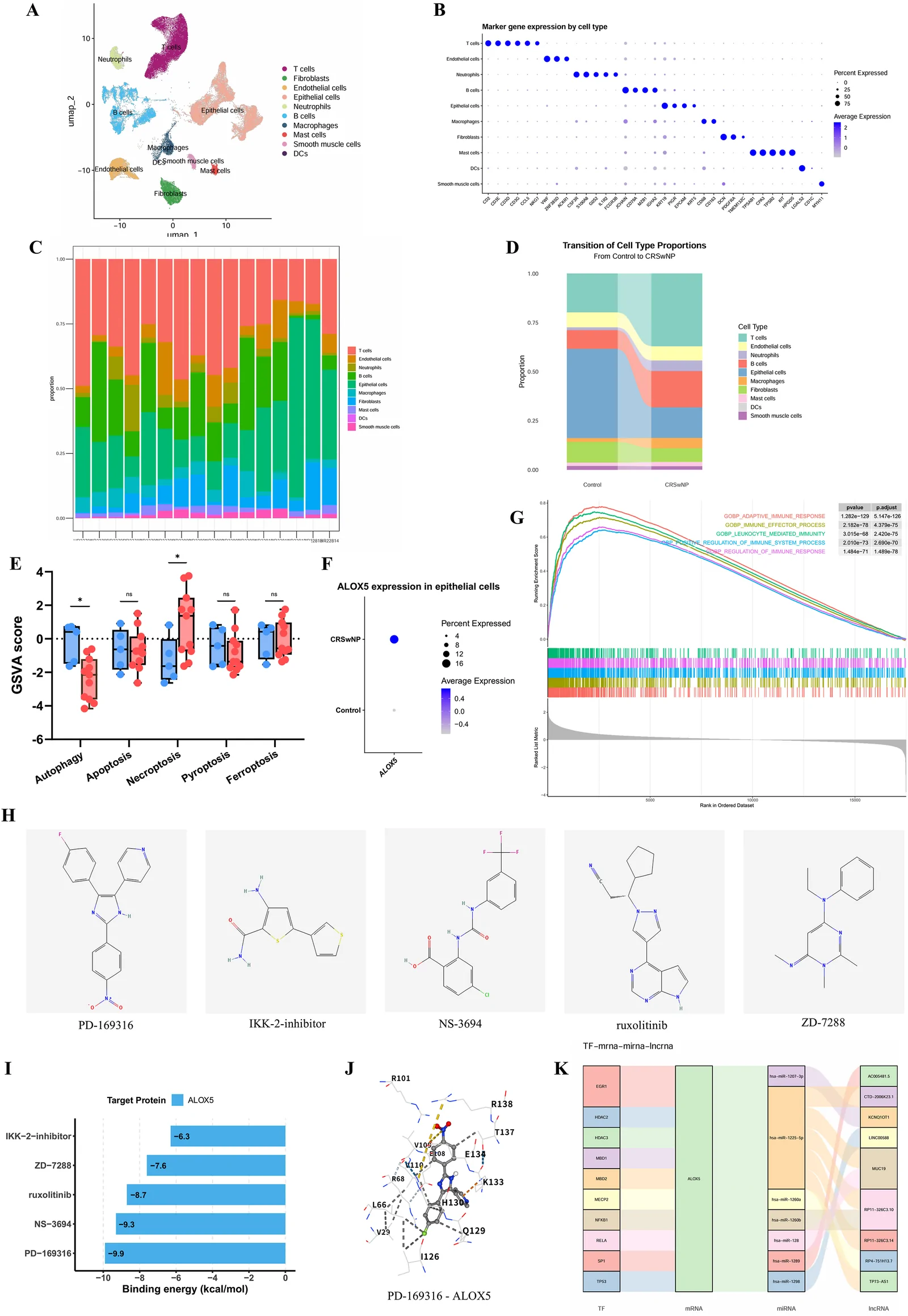
**Single-cell and molecular analyses of ALOX5 in CRSwNP**. (A) UMAP dimensionality reduction and clustering analysis of cells from all samples. (B) Bubble plot illustrating the expression of marker genes across different cell types. (C) Stacked bar plot depicting the infiltration of different cell types in each sample. (D) Comparison of cell type proportions between control and CRSwNP groups. (E) Gene set variation analysis (GSVA) of different cell death pathways in nasal epithelial cells from CRSwNP patients and controls, based on single-cell RNA sequencing data (HRA000772). Samples included 5 healthy nasal mucosa and eleven nasal polyps. (F) Expression of ALOX5 in nasal epithelial cells from CRSwNP patients and controls at the single-cell level. (G) Gene set enrichment analysis (GSEA) of ALOX5. (H) Chemical structures of potential small-molecule compounds. (I) Bar plot showing the minimum binding energies obtained from molecular docking. (J) Molecular docking results of PD-169316 with ALOX5. (K) Sankey diagram illustrating the transcription factor (TF)-mRNA-miRNA-lncRNA regulatory network of ALOX5. Significance levels: ∗*P* < 0.05; ∗∗*P* < 0.01; ∗∗∗*P* < 0.001; ∗∗∗∗*P* < 0.0001; ns, not significant.

Single-cell RNA sequencing analysis was conducted to further elucidate the role of ALOX5. After stringent quality control, normalization, and unsupervised dimensionality reduction followed by clustering, a total of 10 major cell types were identified, including neutrophils, macrophages, and epithelial cells (Fig. 6A and B). The corresponding marker genes used for cell type identification are summarized in Supplementary Table S5. A heatmap illustrating the top 10 most significantly expressed genes within each cell subtype is presented in Supplementary Fig. S2E.

The cellular composition of each patient sample was analyzed, and the proportional distribution of the 10 cell types across different samples was visualized using stacked bar plots (Fig. 6C). Further comparison of cell proportions between the Control and CRSwNP groups revealed that the proportions of T cells, B cells, macrophages, and neutrophils were significantly increased in CRSwNP, whereas the proportion of epithelial cells was markedly decreased (Fig. 6D).

Subsequently, GSVA analysis was conducted on nasal epithelial cells to assess the activity of various cell death modalities. The results indicated that, compared with the Control group, nasal epithelial cells from CRSwNP patients exhibited significantly lower autophagy scores and markedly higher ferroptosis scores, whereas no significant differences were observed in apoptosis, necroptosis, or pyroptosis scores between the 2 groups (Fig. 6E). The gene sets used for GSVA analysis of cell death pathways are summarized in Supplementary Table S6.

At the single-cell level, nasal epithelial cells from CRSwNP and Control groups were analyzed, and ALOX5 expression was found to be significantly upregulated in the CRSwNP group (Fig. 6F). GSEA results demonstrated that ALOX5 was predominantly enriched in immune-related pathways, including the adaptive immune response and immune effector processes. These findings suggest that ALOX5 may play a critical role in the immune regulation of CRSwNP (Fig. 6G). Given that ALOX5 is a key enzyme in lipid metabolism that links inflammatory signaling with lipid peroxidation processes, its enrichment in immune-related pathways may reflect its upstream role in immune activation, which can subsequently influence downstream epithelial cell fate.

ALOX5 has been reported to play a significant role in epithelial cell injury across various diseases. Recent studies have demonstrated that ALOX5 plays a pivotal role in renal tubular epithelial cell injury through the regulation of ferroptosis. Consistent with these findings, KEGG analysis revealed that DEERGs were significantly enriched in the ferroptosis pathway (Fig. 3D). Taken together, these findings suggest that ALOX5 may be involved in ferroptosis in nasal epithelial cells and potentially serve as a molecular link between immune-related signaling and ferroptotic processes. However, the precise mechanisms underlying its role in CRSwNP remain to be further elucidated.

### Drug interaction analysis and molecular regulatory network of ALOX5

Connectivity Map (CMap) analysis was performed to identify potential therapeutic compounds targeting CRSwNP. Small-molecule compounds with lower CMap scores were considered more likely to possess therapeutic potential for CRSwNP. As shown in Supplementary Table S7, PD-169316, IKK-2 inhibitor, NS-3694, ruxolitinib, and ZD-7288 were identified as the top 5 small-molecule compounds with the lowest CMap scores associated with ALOX5 (Fig. 6H).

In addition, molecular docking analysis was performed to predict the potential binding sites of these compounds. Among these compounds, PD-169316 exhibited the strongest binding affinity toward ALOX5 (Fig. 6I). Subsequently, the molecular docking conformations were visualized using PyMOL software (Fig. 6J).

To further elucidate how ALOX5 functions in CRSwNP, a comprehensive regulatory network centered on ALOX5 was constructed. Using multiple databases, a regulatory network comprising the transcription factor (TF)–mRNA–miRNA–lncRNA axis was established (Fig. 6K).

### Upregulation of ALOX5 is associated with disruption of epithelial tight junctions in CRSwNP

**Fig. 7:**
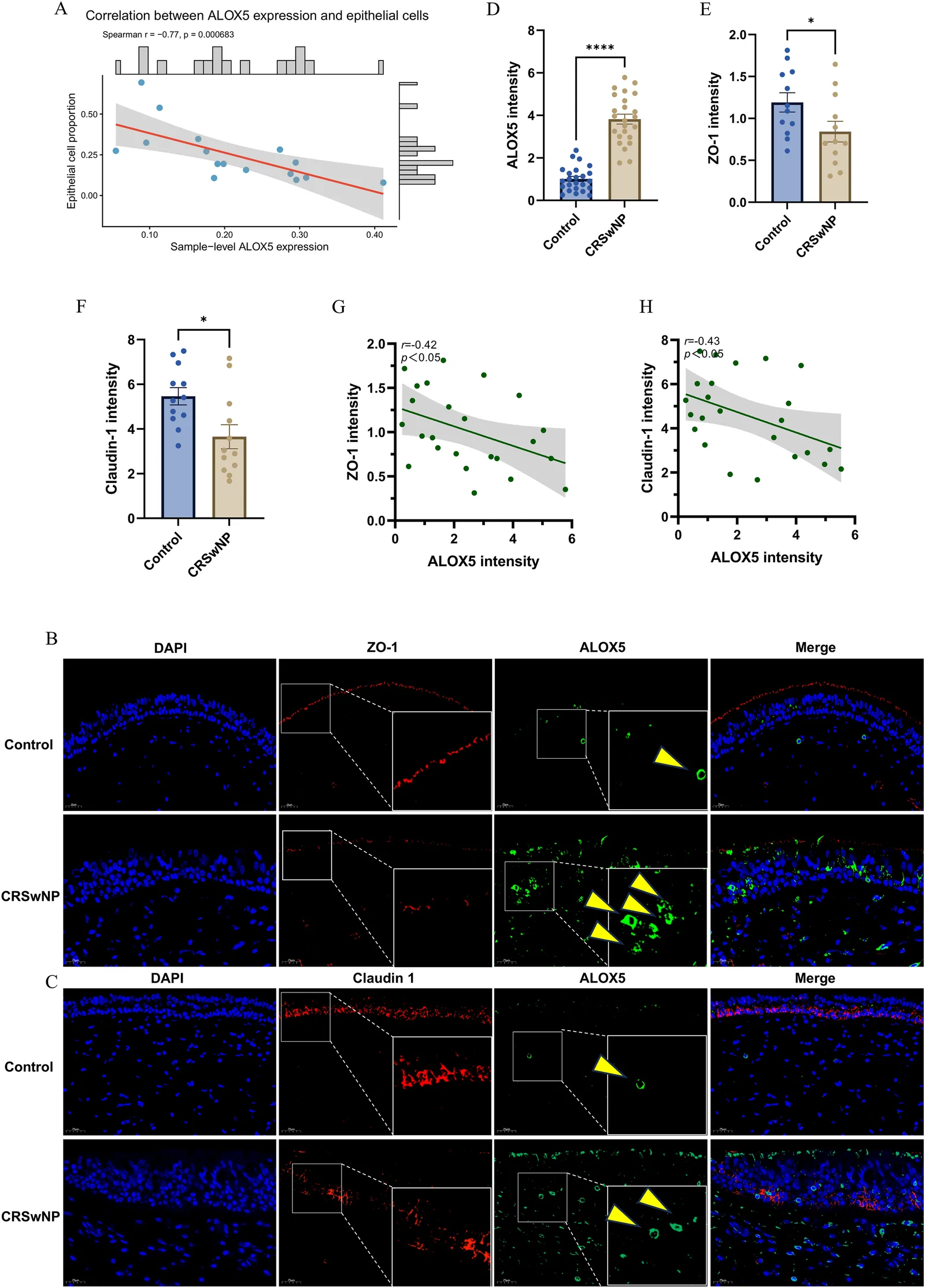
**Single-cell and immunofluorescence analysis of ALOX5 and epithelial tight junction proteins in CRSwNP**. (A) Single-cell RNA sequencing analysis demonstrated a correlation between ALOX5 expression levels and epithelial cell abundance. (B) Immunofluorescence staining was conducted to examine the localization and distribution of ZO-1 in the nasal mucosal epithelium of control and CRSwNP tissues. Scale bar: 25 μm; magnification: 400×. (C) Immunofluorescence staining was performed to evaluate the localization and distribution of Claudin-1 in basal cells. Scale bar: 25 μm; magnification: 400×. (D–F) Quantitative analysis of immunofluorescence intensity of ALOX5 (D), ZO-1 (E), and Claudin-1 (F) in CRSwNP and control tissues. (G–H) Correlation analysis revealed significant negative correlations between ALOX5 expression and ZO-1 (G) as well as Claudin-1 (H). Pearson correlation analysis was performed, with r representing the correlation coefficient. Bar graphs represent the mean ± SD. Significance levels: ∗*P* < 0.05; ∗∗*P* < 0.01; ∗∗∗*P* < 0.001; ∗∗∗∗*P* < 0.0001; ns, not significant.

Single-cell transcriptomic analysis revealed that ALOX5 expression was significantly negatively correlated with epithelial cell abundance (*r* = −0.77, *P* < 0.05; Fig. 7A), suggesting that its upregulation may be associated with epithelial damage.

Immunofluorescence co-localization analysis indicated that epithelial injury in CRSwNP patients was characterized by ciliary loss and abnormal epithelial remodeling, accompanied by goblet cell hyperplasia. In contrast, in the Control group, ZO-1 was uniformly expressed and localized to the apical surface of the nasal epithelium, thereby maintaining intercellular barrier integrity and preventing increased epithelial permeability (Fig. 7B). Claudin-1 was almost exclusively localized to basal cells, where it anchors these cells to the basement membrane and prevents pathogen invasion into the subepithelial compartment (Fig. 7C).

Compared with controls, the fluorescence signals of ZO-1 and Claudin-1 were significantly decreased in CRSwNP tissues (Fig. 7D and E). In contrast, ALOX5 fluorescence intensity was significantly increased in CRSwNP samples (Fig. 7F). Correlation analysis based on quantitative fluorescence data demonstrated that ALOX5 expression was significantly negatively correlated with ZO-1 (*r* = −0.42, *P* < 0.05; Fig. 7G) and Claudin-1 (*r* = −0.43, *P* < 0.05; Fig. 7H). These findings suggest that upregulation of ALOX5 may contribute to the disruption of epithelial tight junction integrity in CRSwNP.

### ALOX5 deficiency suppresses RSL3-Induced ferroptosis in primary human nasal epithelial cells (pHNECs)

**Fig. 8:**
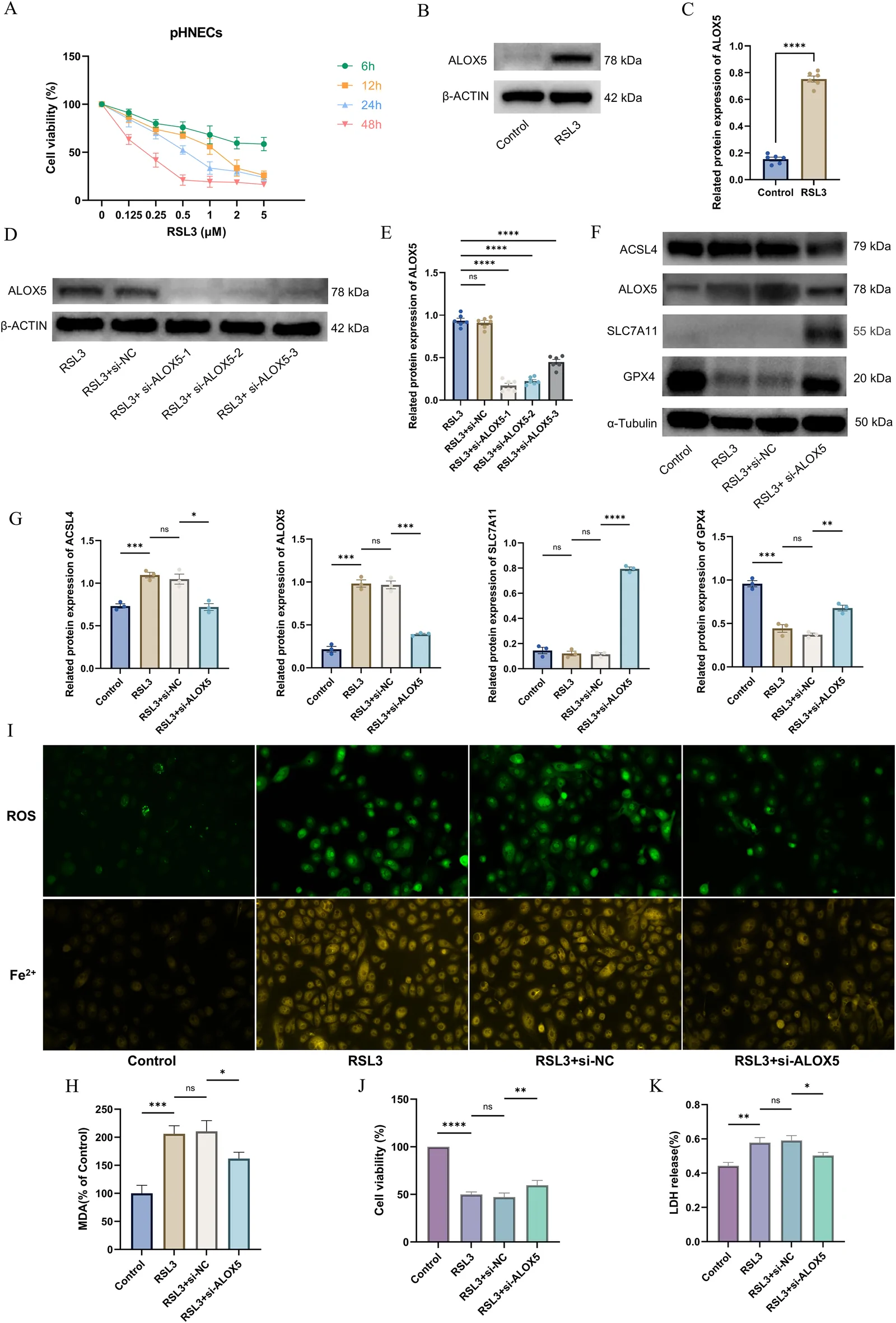
**Effects of ALOX5 silencing on RSL3-induced ferroptosis-related alterations in pHNECs**. (A) Cell viability of pHNECs following treatment with different concentrations of RSL3. (B–C) ALOX5 protein expression in pHNECs after RSL3 treatment was detected by Western blotting, followed by quantitative analysis. (D–E) Transfection efficiency of si-ALOX5 was validated by Western blotting, followed by quantitative analysis. (F–G) Expression levels of ferroptosis-related proteins (GPX4, ACSL4, and SLC7A11) under RSL3 treatment and ALOX5 silencing conditions were detected by Western blotting, followed by quantitative analysis. (H) Malondialdehyde (MDA) levels were measured to assess lipid peroxidation. (I) Immunofluorescence staining was performed to detect Fe^2+^ accumulation and reactive oxygen species (ROS) generation in pHNECs. Magnification: 200×. (J) Cell viability was assessed using the CCK-8 assay. (K) Lactate dehydrogenase (LDH) release assay was performed to evaluate cell membrane damage.Bar graphs represent the mean ± SD.Significance levels: ∗*P* < 0.05; ∗∗*P* < 0.01; ∗∗∗*P* < 0.001; ∗∗∗∗*P* < 0.0001; ns, not significant.

pHNECs were exposed to a range of RSL3 concentrations for varying durations to assess cytotoxicity. As shown in Fig. 8A and Supplementary Fig. S2F, RSL3 induced a dose- and time-dependent decrease in cell viability, with the most pronounced effect observed at 24 h. 0.5 μM RSL3 was selected based on dose–response analysis as an effective concentration capable of inducing ferroptosis while minimizing non-specific cytotoxicity. Accordingly, a 24-h exposure to 0.5 μM RSL3 was applied in subsequent experiments to ensure robust ferroptosis induction while maintaining experimental feasibility.

Exposure to RSL3 markedly increased ALOX5 expression in pHNECs, as demonstrated by Western blot analysis (Fig. 8B and C). Efficient silencing of ALOX5 was achieved using si-ALOX5-1, which exhibited the highest knockdown efficiency among the tested constructs (Fig. 8D and E).

RSL3 stimulation significantly suppressed GPX4 expression while increasing ACSL4 levels, indicating activation of ferroptosis. Notably, ALOX5 silencing attenuated these changes, with a partial restoration of GPX4 expression and a reduction in ACSL4 levels. This suggests that cellular susceptibility to RSL3-induced ferroptosis may depend not only on GPX4 inhibition itself but also on the upstream lipid metabolic context regulated by ALOX5, which may determine the availability of peroxidation-prone lipid substrates. Among the ferroptosis-associated proteins examined, SLC7A11 exhibited the most pronounced expression change, suggesting that it may serve as a critical downstream mediator of ALOX5-driven ferroptosis (Fig. 8F and G).

Having established that ALOX5 silencing reverses RSL3-induced dysregulation of ferroptosis-related proteins, the functional effects of ALOX5 knockdown were further evaluated under conditions simulating oxidative stress and iron homeostasis imbalance in CRSwNP epithelial cells, including lipid peroxidation, oxidative stress, and cell injury outcomes. Malondialdehyde (MDA) measurements revealed that RSL3 treatment significantly increased lipid peroxidation in pHNECs, whereas ALOX5 knockdown markedly attenuated RSL3-induced MDA elevation (Fig. 8H). Consistently, immunofluorescence analysis demonstrated that RSL3 stimulation markedly promoted Fe^2+^ accumulation and reactive oxygen species (ROS) generation in pHNECs, which were significantly alleviated upon ALOX5 silencing (Fig. 8I). Furthermore, cell viability was assessed using the CCK-8 assay, and cell membrane integrity was evaluated via lactate dehydrogenase (LDH) release assay. Results indicated that RSL3 treatment significantly decreased cell viability and increased LDH release in pHNECs, whereas ALOX5 knockdown significantly enhanced cell survival and reduced LDH release (Fig. 8J and K). Collectively, these findings indicate that ALOX5 silencing protects pHNECs from RSL3-induced ferroptotic cell death by suppressing oxidative stress and lipid peroxidation, thereby preserving cellular integrity.

## Discussion

Chronic rhinosinusitis with nasal polyps (CRSwNP) is a highly heterogeneous chronic inflammatory disorder characterized by persistent mucosal inflammation, epithelial barrier dysfunction, and aberrant tissue remodeling.^1^ Persistent inflammation and oxidative stress disrupt intracellular homeostasis, among which endoplasmic reticulum stress (ERS) represents a critical adaptive stress response. ERS refers to a cellular stress response triggered by the disruption of protein-folding homeostasis within the endoplasmic reticulum under various pathological or physiological conditions, including oxidative stress, hypoxia, infection, or inflammatory stimulation.^40^

Recent studies have demonstrated that ERS serves not only as an essential mechanism for cellular adaptation to environmental changes but is also closely associated with immune regulation and inflammatory responses. Various inflammatory signals can directly induce activation of the unfolded protein response (UPR).^41^ In addition, ERS-associated sensors participate in regulating the differentiation and function of multiple immune cell subsets, including B cells, T cells, dendritic cells, and eosinophils, thereby modulating the inflammatory microenvironment^42^^,^^43^. Notably, accumulating evidence has reported aberrant expression of ERS-related molecules and activation of the UPR pathway in CRSwNP tissues, suggesting that ERS may contribute to the maintenance of inflammation and epithelial dysfunction in this disease^44^, ^45^, ^46^. Given the persistent inflammatory milieu and enhanced oxidative stress observed in the nasal mucosa of CRSwNP, ERS-related genes are hypothesized to play regulatory roles in disease-associated transcriptional reprogramming. Based on this biological rationale, ERS-related gene sets were employed as a functional filtering dimension, and a multi-layered integrative analytical framework was constructed to identify potential key regulators enriched within complex transcriptomic alterations under stress-related contexts.

Weighted Gene Co-expression Network Analysis (WGCNA) was first performed to identify co-expression modules significantly associated with CRSwNP. These modules were intersected with differentially expressed genes and an ERS-related gene set, yielding 26 differentially expressed DEERGs. Subsequently, PPI network analysis was conducted to further refine these candidates into 14 potential hub genes. To enhance robustness and minimize model-specific bias, 3 independent machine learning algorithms—LASSO regression, SVM-RFE, and random forest—were applied for consensus feature selection. This integrative strategy identified 4 hub genes—ALOX5, CARD9, HMOX1, and PXDN—with ALOX5 exhibiting the strongest correlation with clinical severity indices, including Lund–Mackay and Lund–Kennedy scores. The application of machine learning in biomarker discovery has been shown to enhance reproducibility and reduce false positives, particularly when integrating multiple approaches; this has been demonstrated in studies of diverse chronic inflammatory conditions and cancer biomarker discovery^47^, ^48^, ^49^.

ALOX5 encodes arachidonate 5-lipoxygenase, a key enzyme in leukotriene biosynthesis and a major mediator of lipid peroxidation.^50^ Leukotrienes are potent inflammatory mediators implicated in asthma and chronic airway inflammation, and activation of the ALOX5 pathway has been associated with enhanced neutrophilic inflammation and oxidative stress in respiratory disease models.^51^ Given the central role of lipid mediators in orchestrating inflammatory responses, ALOX5 may serve as a molecular link between metabolic dysregulation, immune activation, and epithelial injury.

Notably, single-cell and enrichment analyses identified distinct immune- and inflammation-related pathway signatures associated with ALOX5. ALOX5 is classically recognized as an enzyme predominantly involved in immune and inflammatory processes, particularly in regulating lipid mediator biosynthesis in airway inflammatory diseases. In this context, the immune-related signatures observed in our single-cell analysis are consistent with its established biological functions and likely reflect the complex inflammatory microenvironment characteristic of CRSwNP rather than cell type–restricted mechanisms. Given that epithelial dysfunction and barrier remodeling have been established as core pathological features of CRSwNP, we further investigated whether ALOX5 is also involved in local epithelial pathology. Single-cell analysis further demonstrated that ALOX5 exhibited significantly differential expression patterns in nasal epithelial cell populations between CRSwNP patients and controls, suggesting that ALOX5 may exert not only tissue-level inflammatory regulatory functions but also a compartment-specific functional role within the epithelial niche.

Therefore, we employed primary human nasal epithelial cells *in vitro*, which were designed as a focused mechanistic approach to delineate the direct biological effects of epithelial-derived ALOX5 under controlled conditions. This experimental design enables functional dissection of epithelial ALOX5 activity independent of its broader immune-context signaling, thereby allowing compartment-specific mechanistic interpretation.

In this context, elevated ALOX5 expression in CRSwNP may promote the generation of pro-inflammatory lipid species, thereby exacerbating redox imbalance and facilitating regulated cell death pathways. Dysregulation of lipid metabolism and lipid peroxidation in the CRSwNP epithelium has been documented, with elevated lipid peroxidation signals predominantly localized to the epithelial regions of nasal polyps and associated with epithelial dysfunction and inflammation^52^^,^^53^.

Mechanistically, ALOX5-mediated arachidonic acid metabolism may represent a shared substrate axis linking leukotriene biosynthesis and lipid peroxidation processes. ALOX5 catalyzes arachidonic acid utilization to generate downstream leukotriene metabolites, which are central mediators of inflammatory signaling pathways, while simultaneously reshaping the availability and composition of oxidation-prone lipid substrates.^54^ In parallel, increasing evidence suggests that ALOX5 can directly participate in enzymatic lipid oxidation through generation of lipid hydroperoxides, thereby amplifying lipid peroxidation and sensitizing cells to ferroptotic injury.^55^ In addition, emerging evidence indicates that ALOX5 participates not only in inflammation-related disorders but has also been implicated in regulating lipid peroxidation during ferroptosis across multiple disease contexts^56^, ^57^, ^58^ ALOX5 inhibition protects. Given that ferroptosis is characterized by iron-dependent accumulation of lipid peroxides and that ALOX5 catalyzes the oxidation of polyunsaturated fatty acids, aberrant ALOX5 expression may enhance oxidative stress and lipid peroxidation. Considering the persistent inflammation and heightened oxidative stress observed in CRSwNP tissues, ALOX5 is hypothesized to function as a bridging mediator between inflammatory amplification and oxidative injury.

Single-cell transcriptomic analysis further elucidated the pathological mechanisms of epithelial cells in CRSwNP. Compared with healthy controls, epithelial cells from CRSwNP exhibited significantly elevated ferroptosis scores, suggesting a critical role of ferroptosis in epithelial injury and mucosal barrier disruption. Additionally, ALOX5 expression was negatively correlated with epithelial cell abundance, indicating that its overactivation may exacerbate epithelial injury via lipid peroxidation, resulting in compromised barrier function. Overall, these findings indicate that ALOX5-mediated ferroptosis may represent a key mechanism underlying epithelial barrier disruption in CRSwNP and provide a potential target for therapeutic intervention.

Based on these findings, functional validation experiments were performed in primary human nasal epithelial cells (pHNECs). Results showed that ALOX5 knockdown attenuated RSL3-induced ferroptosis, restored GPX4 expression, suppressed ACSL4, upregulated SLC7A11, reduced lipid peroxidation (assessed via MDA levels), decreased iron accumulation and ROS generation, and enhanced cell viability. These data suggest that ALOX5 modulates cellular susceptibility to RSL3-induced ferroptosis, rather than functioning solely as a direct upstream regulator of GPX4. This modulatory effect may reflect an ALOX5-dependent lipid metabolic environment that influences the availability of lipid substrates susceptible to peroxidation and/or promotes a positive feedback loop that amplifies lipid peroxidation signaling^59^, ^60^, ^61^. Therefore, ALOX5 is likely to function as a critical regulatory node governing ferroptotic susceptibility in nasal epithelial cells through context-dependent coordination of lipid peroxidation and iron metabolism–related pathways.

Epithelial barrier dysfunction is a hallmark of CRSwNP and is associated with reduced expression of tight junction proteins, such as ZO-1 and Claudin-1, increased epithelial permeability, and heightened susceptibility to pathogen invasion. In our study, elevated ALOX5 levels in polyp tissues were negatively correlated with ZO-1 and Claudin-1 expression and were accompanied by morphological features of epithelial remodeling, including ciliary loss and goblet cell hyperplasia. Lipid peroxidation products can directly disrupt phospholipid membranes and tight junction complexes, thereby exacerbating the loss of barrier integrity. Collectively, ALOX5-mediated ferroptosis may represent a critical pathogenic nexus linking metabolic dysregulation to structural epithelial injury in CRSwNP.

Notably, molecular docking analysis demonstrated that PD-169316 exhibited the strongest binding affinity toward ALOX5. PD-169316 is a selective inhibitor of p38 MAPK, a signaling pathway that plays a central role in inflammatory signaling, oxidative stress responses, and endoplasmic reticulum stress–related pathways^62^^,^^63^. The predicted binding of PD-169316 to ALOX5 raises the possibility that this compound may possess additional pharmacological interactions beyond its established p38 inhibitory activity. Although ALOX5 has traditionally been regarded as a key enzyme in the leukotriene pathway, accumulating evidence indicates that it may also participate in lipid peroxidation and stress-related inflammatory amplification^64^^,^^65^. Therefore, pharmacological targeting of ALOX5 may represent a potential therapeutic strategy for CRSwNP.

Several limitations of this study should be acknowledged. First, although ALOX5 was consistently associated with clinical severity and mechanistic indicators, larger prospective clinical cohorts are required to validate its diagnostic and prognostic performance. Second, the upstream regulatory signals governing ALOX5 expression in CRSwNP remain to be elucidated; type 2 cytokines, oxidative stress, and metabolic cues may collectively contribute to its induction. Third, *in vivo* models are needed to determine whether pharmacological inhibition of ALOX5 or modulation of ferroptosis can ameliorate polyp formation and epithelial injury without inducing off-target effects. Finally, healthy control tissues were obtained from the inferior turbinate region, whereas nasal polyp tissues were collected from the middle meatus of patients with CRSwNP. Although this sampling strategy has been widely adopted because obtaining anatomically matched healthy sinonasal tissues is ethically and practically challenging, anatomical and spatial transcriptomic differences between tissue sites may introduce potential bias and should therefore be considered when interpreting the findings.

In conclusion, this study integrated multi-omics analyses with experimental validation and identified ALOX5 as a key mediator linking dysregulated oxidative lipid metabolism, ferroptosis, and epithelial barrier dysfunction in CRSwNP. These findings provide mechanistic insight into how aberrant lipid peroxidation drives mucosal injury and chronic inflammation and highlight ALOX5 as a promising target for future therapeutic development.

## Conclusion

In summary, this study systematically identified key genes closely associated with CRSwNP through integrative bioinformatics analyses and experimental validation, established a multi-layered analytical framework centered on endoplasmic reticulum stress as a functional dimension, and ultimately defined ALOX5, CARD9, HMOX1, and PXDN as potential core regulatory factors. Among these, ALOX5 was not only significantly associated with disease severity but also played a pivotal regulatory role in lipid peroxidation, ferroptosis, and epithelial barrier injury, potentially serving as a central molecular hub linking metabolic dysregulation to chronic inflammatory amplification.Furthermore, integration of single-cell transcriptomic analysis with *in vitro* functional assays further elucidated the potential mechanisms by which ALOX5 promotes ferroptosis and epithelial dysfunction, and molecular docking analysis predicted candidate small molecules targeting this pathway, thereby providing novel therapeutic prospects for precision intervention in CRSwNP.

## Ethics statement

This study was approved by the Ethics Committee of the Eye, Ear, Nose and Throat Hospital of Fudan University (Approval No. 2025022).

## CRediT authorship contribution statement

**Ziheng Huang:** Writing – original draft, Visualization, Software, Methodology, Investigation, Formal analysis, Data curation, Conceptualization. **Huiqin Zhou:** Writing – review & editing, Resources, Methodology, Funding acquisition, Data curation, Conceptualization. **Li Wang:** Visualization, Methodology, Investigation. **Yuanyuan Yang:** Software, Conceptualization. **Xiaoqiang Chen:** Software, Data curation. **Chengxun Li:** Software, Data curation. **Hongmeng Yu:** Writing – review & editing, Resources, Project administration, Funding acquisition, Conceptualization. **Desheng Wang:** Writing – review & editing, Supervision, Project administration.

## Data availability

Single-cell RNA-seq data used in this study were obtained from the GSA (HRA000772) and the GEO database (GSE136825, GSE72713, and GSE179265).

## Disclosure statement regarding generative AI and AI-assisted technologies

Nothing to disclose.

## Funding

This work was supported by the 10.13039/501100001809National Natural Science Foundation of China (82301276, 82371123); the 10.13039/501100003399Shanghai Science and Technology Committee Foundation (23Y31900500); the New Technologies of Endoscopic Surgery in Skull Base Tumor: CAMS Innovation Fund for Medical Sciences (CIFMS) (2019-I2M-5-003); the Shanghai Young Scientific and Technological Talents Sailing Program (23YF1404800); the Xuhui Science and Technology Committee Foundation (25XHYD-24); the Municipal Hospital Joint Research Project on Emerging and Cutting-edge Technologies of Shanghai Shenkang Hospital Development Center（SHDC12025122）

## Declaration of competing interest

The authors declare that they have no known competing financial interests or personal relationships that could have appeared to influence the work reported in this paper.
