# Supplementary material for: Multi-omics and machine learning identify ALOX5 as a ferroptosis-associated driver of epithelial barrier dysfunction in chronic rhinosinusitis with nasal polyps
Source: World Allergy Organ J. 2026 Sep 7;19(9):101456. doi: 10.1016/j.waojou.2026.101456 (PMC13578981; doi:10.1016/j.waojou.2026.101456)
Supplement: Supplementary file 1 [file mmc1.docx]

**Supplementary Fig. S1. Data preprocessing and differential expression analysis.**

(A) Principal component analysis (PCA) illustrating batch effects in the integrated dataset before and after batch effect correction. (B) Heatmap showing the expression patterns of DEGs between CRSwNP and control groups. (C) Disease Ontology (DO) enrichment analysis of DEERGs.

**Supplementary Fig. S2. Single-cell and molecular analyses of ALOX5 in CRSwNP.**

(A) LASSO coefficient profiles showing the changes in gene coefficients across different penalty parameters (λ). (B) Random forest analysis: Eight key genes were identified from the core genes using the random forest (RF) algorithm. (C) Expression distribution analysis: Density distribution plots illustrating the expression patterns of hub genes in CRSwNP and control groups. (D) External validation: Expression differences of hub genes were validated in the independent GEO dataset GSE179265. (E) Heatmap showing the top 10 differentially expressed genes in each cell type. (F) Dose– and time–response curves and IC50 analysis of RSL3 in primary human nasal epithelial cells (pHNECs).
