# Supplementary material for: Multi-omics and machine learning identify ALOX5 as a ferroptosis-associated driver of epithelial barrier dysfunction in chronic rhinosinusitis with nasal polyps
Source: World Allergy Organ J. 2026 Sep 7;19(9):101456. doi: 10.1016/j.waojou.2026.101456 (PMC13578981; doi:10.1016/j.waojou.2026.101456)
Supplement: Supplementary file 2 [file mmc2.docx]

Supplementary Table S1. Characteristics of subjects

| Variable | Control | CRSwNP |
| --- | --- | --- |
| Total no. of subjects | 15 | 30 |
| Tissue used | IT | NP |
| Age (y), mean (SD) | 38.15(14.72) | 43.98(13.60) |
| Male,NO.(%) | 11(73.33%) | 17(56.67%) |
| Asthma, (No.) | 0 | 0 |
| Aspirin sensitivity (No.) | 0 | 0 |
| Nasal steroid, (No.) | 0 | 0 |
| Smoker,NO.(%) | 4(26.67%) | 13(43.34%) |
| Lund-Kennedy score, mean (SD) | - | 13.07 (2.73) |
| Lund-Mackay score, mean (SD) | - | 10.73 (4.63) |
| Methodologies | - | - |
| RT-qPCR | 13 | 20 |
| Western blotting | 9 | 9 |
| Immunohistochemistry | 10 | 10 |
| Immunofluorescence | 24 | 24 |

CRSwNP, chronic rhinosinusitis with nasal polyps; RT-qPCR, reverse transcription quantitative polymerase chain reaction

Supplementary Table S2. The list of primer sequences

| Gene | Forward primer (5′ → 3′) | Reverse primer (5′ → 3′) |
| --- | --- | --- |
| ALOX5 | CTCAAGCAACACCGACGTAAA | CCTTGTGGCATTTGGCATCG |
| CARD9 | ATGTCGGACTACGAGAACGAT | TGATGCGTGAGGGGTCGAT |
| HMOX1 | AAGACTGCGTTCCTGCTCAAC | AAAGCCCTACAGCAACTGTC |
| PXDN | GCTCAGGCACTCAGATGACA | CTTCCCCCTTTGGCTTAGAC |

| Supplementary Table S3: The module genes of CRSwNP | |
| --- | --- |
| Genes | Module |
| ABCC9 | cyan |
| ABI3 | cyan |
| ACAA2 | cyan |
| ACP5 | cyan |
| ADA | cyan |
| ADA2 | cyan |
| ADAM19 | cyan |
| ADAM33 | cyan |
| ADAM8 | cyan |
| ADAMDEC1 | cyan |
| ADAMTS12 | cyan |
| ADAMTS15 | cyan |
| ADAMTS2 | cyan |
| ADAMTSL4 | cyan |
| ADAP2 | cyan |
| ADCY7 | cyan |
| ADGRE2 | cyan |
| ADGRG5 | cyan |
| ADORA2A | cyan |
| ADORA3 | cyan |
| ADRA2A | cyan |
| AEBP1 | cyan |
| AIF1 | cyan |
| AIM2 | cyan |
| AK5 | cyan |
| ALOX5 | cyan |
| ALOX5AP | cyan |
| AMPH | cyan |
| ANKRD44 | cyan |
| ANKRD44-IT1 | cyan |
| ANTXR2 | cyan |
| ANTXRLP1 | cyan |
| AOAH | cyan |
| APBA2 | cyan |
| APBB1IP | cyan |
| APELA | cyan |
| ARHGAP15 | cyan |
| ARHGAP25 | cyan |
| ARHGAP30 | cyan |
| ARHGAP4 | cyan |
| ARHGAP45 | cyan |
| ARHGAP9 | cyan |
| ARHGDIB | cyan |
| ARHGEF6 | cyan |
| ARID3A | cyan |
| ARL11 | cyan |
| ARL4C | cyan |
| ARRB2 | cyan |
| ARSB | cyan |
| ATP8B4 | cyan |
| BANK1 | cyan |
| BARX1 | cyan |
| BCL2A1 | cyan |
| BGN | cyan |
| BIN2 | cyan |
| BLK | cyan |
| BNC2 | cyan |
| BTK | cyan |
| BTLA | cyan |
| BTNL12P | cyan |
| C11orf21 | cyan |
| C16orf54 | cyan |
| C19orf38 | cyan |
| C1QA | cyan |
| C1QB | cyan |
| C1QC | cyan |
| C1R | cyan |
| C1S | cyan |
| C1orf162 | cyan |
| C3 | cyan |
| C3AR1 | cyan |
| C4A | cyan |
| C4B | cyan |
| C5AR1 | cyan |
| C5orf58 | cyan |
| CACNA1C | cyan |
| CARD11 | cyan |
| CARD8 | cyan |
| CARD9 | cyan |
| CASS4 | cyan |
| CCDC152 | cyan |
| CCDC8 | cyan |
| CCDC80 | cyan |
| CCL11 | cyan |
| CCL5 | cyan |
| CCN4 | cyan |
| CCND2 | cyan |
| CCR1 | cyan |
| CCR2 | cyan |
| CCR5 | cyan |
| CCR6 | cyan |
| CCR7 | cyan |
| CD163 | cyan |
| CD163L1 | cyan |
| CD180 | cyan |
| CD19 | cyan |
| CD1C | cyan |
| CD1D | cyan |
| CD1E | cyan |
| CD2 | cyan |
| CD209 | cyan |
| CD22 | cyan |
| CD244 | cyan |
| CD247 | cyan |
| CD248 | cyan |
| CD27 | cyan |
| CD28 | cyan |
| CD300A | cyan |
| CD300C | cyan |
| CD300E | cyan |
| CD300LB | cyan |
| CD300LF | cyan |
| CD33 | cyan |
| CD37 | cyan |
| CD3D | cyan |
| CD3E | cyan |
| CD3G | cyan |
| CD4 | cyan |
| CD40LG | cyan |
| CD48 | cyan |
| CD52 | cyan |
| CD53 | cyan |
| CD6 | cyan |
| CD68 | cyan |
| CD72 | cyan |
| CD74 | cyan |
| CD79B | cyan |
| CD84 | cyan |
| CD86 | cyan |
| CD8A | cyan |
| CD8B | cyan |
| CD96 | cyan |
| CDH11 | cyan |
| CEACAM21 | cyan |
| CEACAM3 | cyan |
| CELF2 | cyan |
| CERCAM | cyan |
| CFI | cyan |
| CFP | cyan |
| CHGB | cyan |
| CHN1 | cyan |
| CHRD | cyan |
| CHST11 | cyan |
| CHST15 | cyan |
| CIITA | cyan |
| CKLF | cyan |
| CKLF-CMTM1 | cyan |
| CLDN11 | cyan |
| CLDN5 | cyan |
| CLEC10A | cyan |
| CLEC11A | cyan |
| CLEC12A | cyan |
| CLEC4A | cyan |
| CLEC7A | cyan |
| CLECL1P | cyan |
| CLIP3 | cyan |
| CLMAT3 | cyan |
| CLMP | cyan |
| CMA1 | cyan |
| CMKLR1 | cyan |
| CMTM3 | cyan |
| CNTN4 | cyan |
| CNTN4-AS1 | cyan |
| COL10A1 | cyan |
| COL11A1 | cyan |
| COL12A1 | cyan |
| COL15A1 | cyan |
| COL16A1 | cyan |
| COL1A1 | cyan |
| COL1A2 | cyan |
| COL26A1 | cyan |
| COL3A1 | cyan |
| COL4A1 | cyan |
| COL4A2-AS2 | cyan |
| COL5A1 | cyan |
| COL5A2 | cyan |
| COL6A1 | cyan |
| COL6A2 | cyan |
| COL6A3 | cyan |
| COL8A1 | cyan |
| COL8A2 | cyan |
| COLEC12 | cyan |
| CORO1A | cyan |
| CORO1A-AS1 | cyan |
| CPA3 | cyan |
| CPT1C | cyan |
| CPVL | cyan |
| CPXM1 | cyan |
| CPXM2 | cyan |
| CPZ | cyan |
| CR1 | cyan |
| CRABP2 | cyan |
| CRISPLD2 | cyan |
| CSF1R | cyan |
| CSF2RA | cyan |
| CSF2RB | cyan |
| CSF3R | cyan |
| CSGALNACT2 | cyan |
| CSK | cyan |
| CST7 | cyan |
| CTSG | cyan |
| CTSK | cyan |
| CX3CR1 | cyan |
| CXCL17 | cyan |
| CXCR1 | cyan |
| CXCR2 | cyan |
| CXCR3 | cyan |
| CXCR6 | cyan |
| CYBB | cyan |
| CYBC1 | cyan |
| CYSLTR1 | cyan |
| CYTH3 | cyan |
| CYTH4 | cyan |
| CYTIP | cyan |
| CYTOR | cyan |
| DAB2 | cyan |
| DCN | cyan |
| DIO3OS | cyan |
| DLL4 | cyan |
| DNM1 | cyan |
| DOCK10 | cyan |
| DOCK11 | cyan |
| DOCK2 | cyan |
| DOCK8 | cyan |
| DOCK8-AS1 | cyan |
| DOCK8-AS2 | cyan |
| DOK2 | cyan |
| DOK3 | cyan |
| DPEP2 | cyan |
| DPP6 | cyan |
| DYSF | cyan |
| EDIL3 | cyan |
| EFEMP1 | cyan |
| EFEMP2 | cyan |
| EIF4EBP1 | cyan |
| EMILIN1 | cyan |
| EML4-AS1 | cyan |
| EMP3 | cyan |
| ENPEP | cyan |
| ENPP2 | cyan |
| EVI2A | cyan |
| EVI2B | cyan |
| F13A1 | cyan |
| FADS1 | cyan |
| FADS2 | cyan |
| FAM3B | cyan |
| FAM78A | cyan |
| FAP | cyan |
| FASLG | cyan |
| FBLN1 | cyan |
| FBLN5 | cyan |
| FBN1 | cyan |
| FCER1G | cyan |
| FCER2 | cyan |
| FCGR1A | cyan |
| FCGR2A | cyan |
| FCGR2B | cyan |
| FCGR2C | cyan |
| FCGR3A | cyan |
| FCGR3B | cyan |
| FCGRT | cyan |
| FCHO1 | cyan |
| FCMR | cyan |
| FCN1 | cyan |
| FCRL1 | cyan |
| FCRL2 | cyan |
| FCRL3 | cyan |
| FCRL6 | cyan |
| FCRLA | cyan |
| FERMT3 | cyan |
| FFAR2 | cyan |
| FGD2 | cyan |
| FGD3 | cyan |
| FGF14 | cyan |
| FGF7 | cyan |
| FGL2 | cyan |
| FGR | cyan |
| FKBP10 | cyan |
| FLT3 | cyan |
| FMN2 | cyan |
| FN1 | cyan |
| FNDC1 | cyan |
| FOLR2 | cyan |
| FPR1 | cyan |
| FPR3 | cyan |
| FSTL1 | cyan |
| FSTL3 | cyan |
| FYB1 | cyan |
| FYN | cyan |
| FZD2 | cyan |
| GAB3 | cyan |
| GAPT | cyan |
| GATA2 | cyan |
| GFRA2 | cyan |
| GIMAP1 | cyan |
| GIMAP2 | cyan |
| GJA1 | cyan |
| GJA5 | cyan |
| GLI1 | cyan |
| GLIPR1 | cyan |
| GLT8D2 | cyan |
| GMFG | cyan |
| GMIP | cyan |
| GNAO1 | cyan |
| GNG2 | cyan |
| GPR132 | cyan |
| GPR171 | cyan |
| GPR174 | cyan |
| GPR176 | cyan |
| GPR183 | cyan |
| GPR34 | cyan |
| GPR65 | cyan |
| GPSM1 | cyan |
| GPSM3 | cyan |
| GRAP2 | cyan |
| GREM1 | cyan |
| GREM1-AS1 | cyan |
| GREM2 | cyan |
| GRIA3 | cyan |
| GSC | cyan |
| GSC-DT | cyan |
| GVINP1 | cyan |
| GZMA | cyan |
| GZMH | cyan |
| GZMK | cyan |
| GZMM | cyan |
| HAVCR2 | cyan |
| HCK | cyan |
| HCLS1 | cyan |
| HCST | cyan |
| HDC | cyan |
| HEG1 | cyan |
| HEPH | cyan |
| HK3 | cyan |
| HLA-DMA | cyan |
| HLA-DMB | cyan |
| HLA-DOA | cyan |
| HLA-DPA1 | cyan |
| HLA-DPB1 | cyan |
| HLA-DQB2 | cyan |
| HLA-DRA | cyan |
| HLA-DRB1 | cyan |
| HMCN1 | cyan |
| HMOX1 | cyan |
| HRH1 | cyan |
| HRH2 | cyan |
| HSPA7 | cyan |
| HTRA3 | cyan |
| HVCN1 | cyan |
| ICAM3 | cyan |
| IDO1 | cyan |
| IFFO1 | cyan |
| IGF1 | cyan |
| IGF2 | cyan |
| IGFLR1 | cyan |
| IGSF6 | cyan |
| IKZF1 | cyan |
| IKZF3 | cyan |
| IL10 | cyan |
| IL10RA | cyan |
| IL12RB1 | cyan |
| IL16 | cyan |
| IL21R | cyan |
| IL27RA | cyan |
| IL2RA | cyan |
| IL2RB | cyan |
| IL2RG | cyan |
| IL32 | cyan |
| IL3RA | cyan |
| IL4I1 | cyan |
| IL7R | cyan |
| INHBA | cyan |
| INPP5D | cyan |
| INS-IGF2 | cyan |
| IRAG2 | cyan |
| IRF5 | cyan |
| IRF8 | cyan |
| ISLR | cyan |
| ITGA11 | cyan |
| ITGA4 | cyan |
| ITGAL | cyan |
| ITGAM | cyan |
| ITGAX | cyan |
| ITGB2 | cyan |
| ITGB2-AS1 | cyan |
| ITGB7 | cyan |
| ITGBL1 | cyan |
| ITK | cyan |
| JAK3 | cyan |
| JAML | cyan |
| KCNAB2 | cyan |
| KCNE4 | cyan |
| KCNIP1 | cyan |
| KCNJ8 | cyan |
| KCNJ8-AS1 | cyan |
| KIF26B | cyan |
| KIF5C | cyan |
| KLF12 | cyan |
| KLHDC7B | cyan |
| KLRB1 | cyan |
| KLRC4-KLRK1 | cyan |
| KLRG1 | cyan |
| KLRK1 | cyan |
| KLRK1-AS1 | cyan |
| KMO | cyan |
| LAIR1 | cyan |
| LAMP5 | cyan |
| LAMP5-AS1 | cyan |
| LAPTM5 | cyan |
| LAT | cyan |
| LAT2 | cyan |
| LCK | cyan |
| LCP1 | cyan |
| LCP2 | cyan |
| LGALS1 | cyan |
| LGMN | cyan |
| LHFPL7 | cyan |
| LILRA1 | cyan |
| LILRA5 | cyan |
| LILRA6 | cyan |
| LILRB1 | cyan |
| LILRB2 | cyan |
| LILRB3 | cyan |
| LILRB4 | cyan |
| LILRB5 | cyan |
| LIMD2 | cyan |
| LINC00426 | cyan |
| LINC00861 | cyan |
| LINC01094 | cyan |
| LINC01397 | cyan |
| LINC01934 | cyan |
| LINC03070 | cyan |
| LOC101448202 | cyan |
| LOC101927283 | cyan |
| LOC101927947 | cyan |
| LOC101927954 | cyan |
| LOC102723635 | cyan |
| LOC102724765 | cyan |
| LOC105370003 | cyan |
| LOC105373024 | cyan |
| LOC105373791 | cyan |
| LOC105373905 | cyan |
| LOC105373906 | cyan |
| LOC105374925 | cyan |
| LOC105374981 | cyan |
| LOC105377291 | cyan |
| LOC105377945 | cyan |
| LOC105377979 | cyan |
| LOC105379189 | cyan |
| LOC107984131 | cyan |
| LOC107985020 | cyan |
| LOC107986633 | cyan |
| LOC606724 | cyan |
| LOXL2 | cyan |
| LOXL2-AS1 | cyan |
| LOXL3 | cyan |
| LPCAT1 | cyan |
| LPXN | cyan |
| LRP2 | cyan |
| LRRC25 | cyan |
| LSP1 | cyan |
| LST1 | cyan |
| LTB | cyan |
| LTBP2 | cyan |
| LUM | cyan |
| LY86 | cyan |
| LY9 | cyan |
| LYPD6 | cyan |
| LYVE1 | cyan |
| MAB21L1 | cyan |
| MAP1LC3C | cyan |
| MAP3K5-AS2 | cyan |
| MAP4K1 | cyan |
| MAP4K1-AS1 | cyan |
| MARCHF1 | cyan |
| MARCO | cyan |
| MATN3 | cyan |
| MEF2C | cyan |
| MEF2C-AS2 | cyan |
| MFNG | cyan |
| MGARP | cyan |
| MIAT | cyan |
| MICB | cyan |
| MICOS10-NBL1 | cyan |
| MILR1 | cyan |
| MIR1247 | cyan |
| MIR181A1HG | cyan |
| MIR198 | cyan |
| MIR214 | cyan |
| MIR223 | cyan |
| MIR3120 | cyan |
| MIR3606 | cyan |
| MIR4435-2HG | cyan |
| MIR4649 | cyan |
| MIR5196 | cyan |
| MIR7846 | cyan |
| MIR7847 | cyan |
| MIR7848 | cyan |
| MMD | cyan |
| MMP19 | cyan |
| MMP2 | cyan |
| MNDA | cyan |
| MPEG1 | cyan |
| MPP1 | cyan |
| MRC1 | cyan |
| MRC2 | cyan |
| MS4A1 | cyan |
| MS4A14 | cyan |
| MS4A2 | cyan |
| MS4A4A | cyan |
| MS4A6A | cyan |
| MS4A7 | cyan |
| MSC | cyan |
| MSC-AS1 | cyan |
| MSR1 | cyan |
| MXRA5 | cyan |
| MXRA8 | cyan |
| MYL3 | cyan |
| MYO1F | cyan |
| MYO1G | cyan |
| MYO7A | cyan |
| NAALADL1 | cyan |
| NABP1 | cyan |
| NAPSB | cyan |
| NBL1 | cyan |
| NCAM1 | cyan |
| NCF1 | cyan |
| NCF1B | cyan |
| NCF1C | cyan |
| NCF2 | cyan |
| NCF4 | cyan |
| NCF4-AS1 | cyan |
| NCKAP1L | cyan |
| NFAM1 | cyan |
| NFATC4 | cyan |
| NFE2 | cyan |
| NID1 | cyan |
| NID2 | cyan |
| NKD1 | cyan |
| NLRC4 | cyan |
| NLRP3 | cyan |
| NOX4 | cyan |
| NPL | cyan |
| NR2F1-AS1 | cyan |
| NREP | cyan |
| NRROS | cyan |
| NTAN1 | cyan |
| NTM | cyan |
| NTRK1 | cyan |
| NUP210 | cyan |
| NXPE3 | cyan |
| OLFM2 | cyan |
| OLFML2B | cyan |
| OLFML3 | cyan |
| OSCAR | cyan |
| OTULINL | cyan |
| P2RX1 | cyan |
| P2RX5 | cyan |
| P2RY10 | cyan |
| P2RY13 | cyan |
| P2RY8 | cyan |
| P4HA3 | cyan |
| PAG1 | cyan |
| PAPPA | cyan |
| PAPPA-AS1 | cyan |
| PARP15 | cyan |
| PARVB | cyan |
| PARVG | cyan |
| PCED1B | cyan |
| PCED1B-AS1 | cyan |
| PCOLCE | cyan |
| PCOLCE-AS1 | cyan |
| PDCD1LG2 | cyan |
| PDE7B | cyan |
| PDGFRA | cyan |
| PDGFRB | cyan |
| PHACTR1 | cyan |
| PIEZO2 | cyan |
| PIK3CD | cyan |
| PIK3CG | cyan |
| PIK3R5 | cyan |
| PIK3R6 | cyan |
| PILRA | cyan |
| PIM2 | cyan |
| PKN1 | cyan |
| PLA1A | cyan |
| PLA2G7 | cyan |
| PLCB2 | cyan |
| PLCG2 | cyan |
| PLCL2 | cyan |
| PLD4 | cyan |
| PLEK | cyan |
| PLXNC1 | cyan |
| PODNL1 | cyan |
| POSTN | cyan |
| PPP1R18 | cyan |
| PRAM1 | cyan |
| PREX1 | cyan |
| PRF1 | cyan |
| PRKCB | cyan |
| PRKCQ | cyan |
| PSTPIP1 | cyan |
| PTAFR | cyan |
| PTGDR | cyan |
| PTGS1 | cyan |
| PTH1R | cyan |
| PTP4A3 | cyan |
| PTPN22 | cyan |
| PTPN6 | cyan |
| PTPN7 | cyan |
| PTPRC | cyan |
| PTPRCAP | cyan |
| PVRIG | cyan |
| PXDN | cyan |
| PYHIN1 | cyan |
| RAB3IL1 | cyan |
| RAB42 | cyan |
| RAB8B | cyan |
| RAC2 | cyan |
| RASGRF2 | cyan |
| RASGRP4 | cyan |
| RASL11B | cyan |
| RASSF1-AS1 | cyan |
| RASSF2 | cyan |
| RASSF4 | cyan |
| RASSF5 | cyan |
| RCN3 | cyan |
| RCSD1 | cyan |
| RELT | cyan |
| RESF1 | cyan |
| RFLNB | cyan |
| RGS18 | cyan |
| RGS19 | cyan |
| RHBDF2 | cyan |
| RHEX | cyan |
| RHOF | cyan |
| RHOH | cyan |
| RIN3 | cyan |
| RNASE2 | cyan |
| RNASE6 | cyan |
| RNF150 | cyan |
| RNF166 | cyan |
| ROBO2 | cyan |
| ROBO3 | cyan |
| ROR2 | cyan |
| RSPO1 | cyan |
| RUBCNL | cyan |
| RUNX1-IT1 | cyan |
| S1PR4 | cyan |
| SAMD3 | cyan |
| SAMD5 | cyan |
| SAMSN1 | cyan |
| SASH3 | cyan |
| SCG2 | cyan |
| SCIMP | cyan |
| SCML4 | cyan |
| SDC3 | cyan |
| SELENOP | cyan |
| SELL | cyan |
| SELP | cyan |
| SELPLG | cyan |
| SEMA4D | cyan |
| SEPTIN1 | cyan |
| SEPTIN11 | cyan |
| SEPTIN6 | cyan |
| SERPINA1 | cyan |
| SERPINB9 | cyan |
| SERPINB9-AS1 | cyan |
| SERPINH1 | cyan |
| SFRP2 | cyan |
| SH2D1A | cyan |
| SH2D2A | cyan |
| SH2D3C | cyan |
| SIGLEC1 | cyan |
| SIGLEC10 | cyan |
| SIGLEC10-AS1 | cyan |
| SIGLEC5 | cyan |
| SIGLEC7 | cyan |
| SIGLEC9 | cyan |
| SIRPB1 | cyan |
| SIRPB2 | cyan |
| SIRPG | cyan |
| SIT1 | cyan |
| SIX3-AS1 | cyan |
| SKAP2 | cyan |
| SLA | cyan |
| SLA2 | cyan |
| SLAMF1 | cyan |
| SLAMF6 | cyan |
| SLAMF8 | cyan |
| SLC11A1 | cyan |
| SLC14A2-AS1 | cyan |
| SLC2A5 | cyan |
| SLC2A6 | cyan |
| SLC38A11 | cyan |
| SLC7A7 | cyan |
| SLCO2B1 | cyan |
| SLFN12L | cyan |
| SMAP2 | cyan |
| SMIM35 | cyan |
| SMOC2 | cyan |
| SNAI3 | cyan |
| SNHG28 | cyan |
| SNX10 | cyan |
| SNX10-AS1 | cyan |
| SNX20 | cyan |
| SP110 | cyan |
| SP140 | cyan |
| SPARC | cyan |
| SPI1 | cyan |
| SPIB | cyan |
| SPN | cyan |
| SPOCK1 | cyan |
| SPOCK2 | cyan |
| SPON1 | cyan |
| SRGN | cyan |
| SSH2 | cyan |
| ST6GAL2 | cyan |
| STAB1 | cyan |
| STAMBPL1 | cyan |
| STARD4-AS1 | cyan |
| STARD5 | cyan |
| STAT4 | cyan |
| STAT4-AS1 | cyan |
| STK10 | cyan |
| STMN3 | cyan |
| SULF1 | cyan |
| SUSD3 | cyan |
| SYK | cyan |
| SYT11 | cyan |
| TAGAP | cyan |
| TARP | cyan |
| TASL | cyan |
| TBC1D10C | cyan |
| TBX3 | cyan |
| TBXAS1 | cyan |
| TDO2 | cyan |
| TENM3 | cyan |
| TESPA1 | cyan |
| TFAP2B | cyan |
| TFEC | cyan |
| TGFBI | cyan |
| TGM2 | cyan |
| THBS2 | cyan |
| THEMIS | cyan |
| THEMIS2 | cyan |
| THY1 | cyan |
| TLR10 | cyan |
| TLR4 | cyan |
| TLR7 | cyan |
| TLR8 | cyan |
| TMC8 | cyan |
| TMEM106A | cyan |
| TMEM119 | cyan |
| TMEM130 | cyan |
| TMEM156 | cyan |
| TMEM176A | cyan |
| TMEM176B | cyan |
| TMEM204 | cyan |
| TMEM273 | cyan |
| TMIGD3 | cyan |
| TNFAIP6 | cyan |
| TNFAIP8L2 | cyan |
| TNFRSF10C | cyan |
| TNFRSF1B | cyan |
| TNFRSF6B | cyan |
| TNFSF13B | cyan |
| TNFSF14 | cyan |
| TNFSF8 | cyan |
| TNS3 | cyan |
| TOX | cyan |
| TPBGL-AS1 | cyan |
| TRAC | cyan |
| TRAF1 | cyan |
| TRAF3IP3 | cyan |
| TRAJ32 | cyan |
| TRAJ42 | cyan |
| TRAM2 | cyan |
| TRAPPC3L | cyan |
| TRAT1 | cyan |
| TRAV12-2 | cyan |
| TRAV13-1 | cyan |
| TRAV17 | cyan |
| TRAV8-2 | cyan |
| TRAV8-4 | cyan |
| TRBC1 | cyan |
| TRBC2 | cyan |
| TRBJ2-3 | cyan |
| TRBJ2-7 | cyan |
| TRBV11-2 | cyan |
| TRBV12-3 | cyan |
| TRBV12-4 | cyan |
| TRBV20-1 | cyan |
| TRBV7-2 | cyan |
| TRDC | cyan |
| TREML2 | cyan |
| TRG-AS1 | cyan |
| TRGC1 | cyan |
| TRGC2 | cyan |
| TRGV2 | cyan |
| TRGV3 | cyan |
| TRPM2 | cyan |
| TRPV2 | cyan |
| TSPAN11 | cyan |
| TSPAN32 | cyan |
| TSPAN4 | cyan |
| TYROBP | cyan |
| UBASH3A | cyan |
| UBD | cyan |
| UBL4B | cyan |
| UNC13D | cyan |
| VCAM1 | cyan |
| VCAN | cyan |
| VCAN-AS1 | cyan |
| VIM | cyan |
| VIM-AS1 | cyan |
| VOPP1 | cyan |
| VSIG4 | cyan |
| VSIR | cyan |
| WAS | cyan |
| WDFY4 | cyan |
| WIPF1 | cyan |
| XPNPEP2 | cyan |
| ZAP70 | cyan |
| ZEB2-AS1 | cyan |
| ZNF366 | cyan |
| ZNF594-DT | cyan |
|  |  |

Supplementary Table S4. Lund-Mackay and Lund-Kennedy scores of patients with CRSwNP and the number of PCR cycles for the hub genes

| Patients | ALOX5 | CARD9 | HMOX1 | PXDN | Lund-Mackay  score | Lund-Kennedy  score |
| --- | --- | --- | --- | --- | --- | --- |
| NP1 | 3.387 | 1.225 | 5.806 | 2.639 | 15 | 5 |
| NP2 | 4.317 | 1.368 | 4.848 | 4.377 | 15 | 13 |
| NP3 | 2.014 | 2.433 | 3.830 | 1.892 | 7 | 10 |
| NP4 | 1.094 | 1.673 | 1.513 | 1.064 | 7 | 14 |
| NP5 | 2.235 | 1.089 | 2.155 | 2.770 | 13 | 14 |
| NP6 | 6.063 | 1.685 | 2.903 | 2.514 | 15 | 16 |
| NP7 | 3.411 | 0.694 | 4.916 | 2.497 | 11 | 13 |
| NP8 | 4.170 | 1.030 | 3.289 | 1.414 | 15 | 12 |
| NP9 | 1.414 | 2.743 | 4.469 | 1.858 | 15 | 5 |
| NP10 | 1.404 | 1.047 | 2.479 | 1.722 | 15 | 6 |
| NP11 | 3.249 | 2.706 | 5.464 | 3.929 | 13 | 20 |
| NP12 | 1.717 | 0.336 | 3.272 | 3.742 | 12 | 11 |
| NP13 | 6.880 | 3.870 | 1.030 | 4.265 | 18 | 12 |
| NP14 | 4.899 | 4.415 | 4.539 | 0.390 | 16 | 20 |
| NP15 | 2.350 | 3.369 | 3.018 | 1.331 | 10 | 14 |
| NP16 | 2.162 | 1.990 | 2.060 | 1.639 | 11 | 5 |
| NP17 | 0.988 | 5.667 | 1.487 | 1.768 | 13 | 5 |
| NP18 | 3.122 | 5.107 | 2.416 | 0.825 | 14 | 16 |
| NP19 | 3.100 | 2.223 | 3.765 | 4.933 | 12 | 5 |
| NP20 | 0.656 | 4.120 | 1.696 | 1.540 | 13 | 3 |
| NP21 | 2.235 | 1.778 | 4.127 | 5.637 | 14 | 15 |
| NP22 | 3.758 | 2.204 | 0.949 | 1.821 | 13 | 8 |
| NP23 | 0.959 | 0.578 | 2.742 | 3.850 | 9 | 4 |
| NP24 | 3.411 | 3.249 | 2.488 | 1.952 | 15 | 9 |
| NP25 | 1.094 | 4.627 | 4.213 | 3.106 | 15 | 9 |
| NP26 | 1.414 | 2.056 | 4.098 | 1.098 | 11 | 15 |
| NP27 | 4.408 | 2.603 | 2.878 | 2.211 | 9 | 10 |
| NP28 | 2.085 | 3.605 | 3.824 | 0.220 | 17 | 10 |
| NP29 | 5.352 | 5.502 | 2.454 | 0.572 | 14 | 10 |
| NP30 | 2.346 | 2.479 | 4.042 | 2.819 | 15 | 13 |

Supplementary Table S5. Cell Type Annotation and Corresponding Marker Genes

| Cell type | marker gene |
| --- | --- |
| T cells | "CD2", "CD3D", "CD3E", "CD3G", "CCL5","NKG7", |
| B cells | "MZB1", "JCHAIN", "CD79A", "IGHA2", |
| DC | "LGALS2", "CD1C", |
| Macrophage | "CD68", "CD163", |
| Neutrophils | "FCGR3B", "CSF3R", "S100A8", "G0S2", "IL1R2", |
| Mast Cells | "TPSAB1", "CPA3", "TPSB2", "KIT", "HPGDS", |
| Fibroblast | "TMEM132C", "DCN","PDGFRA", |
| Endothelial cells | "VWF", "ACKR1", "ZNF385D", |
| Smooth muscle cells | "MYH11", |
| Epithelial cells | "KRT5", "KRT19","PIGR", "EPCAM" |

Supplementary Table S6. Gene sets of cell death pathways used for GSVA

|  | Genes |
| --- | --- |
| Autophagy | AMBRA1, ARL13B, ATG10, ATG101, ATG12, ATG13, ATG14, ATG16L1, ATG3, ATG4A, ATG4B, ATG4C, ATG4D, ATG5, ATG7, ATG9A, ATG9B, ATM, BECN1, CETN1, CFTR, CHMP2A, CHMP2B, CHMP3, CHMP4A, CHMP4B, CHMP4C, CHMP6, CHMP7, CSNK2A1, CSNK2A2, CSNK2B, DYNC1H1, DYNC1I1, DYNC1I2, DYNC1LI1, DYNC1LI2, DYNLL1, DYNLL2, EEF1A1, EPAS1, FUNDC1, GABARAP, GABARAPL1, GABARAPL2, GFAP, HBB, HDAC6, HSF1, HSP90AA1, HSP90AB1, HSPA8, IFT88, LAMP2, LAMTOR1, LAMTOR2, LAMTOR3, LAMTOR4, LAMTOR5, MAP1LC3A, MAP1LC3B, MAP1LC3C, MFN1, MFN2, MLST8, MTMR14, MTMR3, MTERF3, MTOR, MVB12A, MVB12B, NBR1, PARK7, PCNT, PEDS1-UBE2V1, PEX5, PGAM5, PIK3C3, PIK3R4, PINK1, PLIN2, PLIN3, PRKAA1, PRKAA2, PRKAB1, PRKAB2, PRKAG1, PRKAG2, PRKAG3, PRKN, RB1CC1, RHEB, RNF103-CHMP3, RPS27A, RPTOR, RRAGA, RRAGB, RRAGC, RRAGD, RSE1, SLC38A9, SQSTM1, SRC, TOMM20, TOMM22, TOMM40, TOMM5, TOMM6, TOMM7, TOMM70, TSC1, TSC2, TSG101, TUBA1A, TUBA1B, TUBA1C, TUBA3C, TUBA3D, TUBA3E, TUBA4A, TUBA8, TUBAL3, TUBB1, TUBB2A, TUBB2B, TUBB3, TUBB4A, TUBB4B, TUBB6, TUBB8, UBA52, UBAP1, UBB, UBC, UBE2N, UBE2V1, ULK1, USP30, UVRAG, VCP, VDAC1, VIM, VPS28, VPS37A, VPS37B, VPS37C, VPS37D, WDR45, WDR45B, WIPI1, WIPI2 |
| Apoptosis | ACTB, ACTG1, AIFM1, AKT1, AKT2, AKT3, APAF1, ATF4, ATM, BAD, BAK1, BAX, BBC3, BCL2, BCL2A1, BCL2L1, BCL2L11, BID, BIRC2, BIRC3, BIRC5, CAPN1, CAPN2, CASP10, CASP12, CASP2, CASP3, CASP6, CASP7, CASP8, CASP9, CFLAR, CHUK, CSF2RB, CTSB, CTSC, CTSD, CTSF, CTSH, CTSK, CTSL, CTSO, CTSS, CTSV, CTSW, CTSZ, CYCS, DAB2IP, DAXX, DDIT3, DIABLO, DFFA, DFFB, EIF2AK3, EIF2S1, ENDOG, ERN1, FADD, FAS, FASLG, FOS, GADD45A, GADD45B, GADD45G, GZMB, HRAS, HRK, HTRA2, IKBKB, IKBKG, IL3, IL3RA, ITPR1, ITPR2, ITPR3, JUN, KRAS, LM, LMNB1, LMNB2, MAP2K1, MAP2K2, MAP3K14, MAP3K5, MAPK1, MAPK10, MAPK3, MAPK8, MAPK9, MCL1, NGF, NFKB1, NFKBIA, NTRK1, NRAS, PARP1, PARP2, PARP3, PARP4, PDPK1, PIK3CA, PIK3CB, PIK3CD, PIK3R1, PIK3R2, PIK3R3, PIDD1, PMAIP1, PRF1, PTPN13, RAF1, RELA, RIPK1, SEPTIN4, SPTA1, SPTAN1, TNF, TNFRSF10A, TNFRSF10B, TNFRSF1A, TNFSF10, TP53, TP53AIP1, TRADD, TRAF1, TRAF2, TUBA1A, TUBA1B, TUBA1C, TUBA3C, TUBA3D, TUBA3E, TUBA4A, TUBA8, TUBAL3, XIAP |
| Ferroptosis | ACSL1, ACSL3, ACSL4, ACSL5, ACSL6, AIFM2, AKR1C1, AKR1C2, AKR1C3, ALOX15, ATG5, ATG7, BACH1, CBS, CHMP5, CHMP6, CISD1, COQ2, CP, CTH, CYBB, DPP4, FDFT1, FTH1, FTL, FTMT, GCH1, GCLC, GCLM, GPX4, GSS, HMGCR, HMOX1, HSPB1, IREB2, LPCAT3, MAP1LC3A, MAP1LC3B, MAP1LC3C, NCOA4, NOX1, NOX4, PCBP1, PCBP2, PHKG2, POR, PRNP, SAT1, SAT2, SLC11A2, SLC1A5, SLC38A1, SLC39A14, SLC39A8, SLC3A2, SLC40A1, SLC7A11, STEAP3, TF, TFRC, TP53, TXNRD1, VDAC2, VDAC3 |
| Necroptosis | AIFM1, ALOX15, BAX, BCL2, BID, BIRC2, BIRC3, CAMK2A, CAMK2B, CAMK2D, CAMK2G, CAPN1, CAPN2, CASP1, CASP8, CHMP1A, CHMP1B, CHMP2A, CHMP2B, CHMP3, CHMP4A, CHMP4B, CHMP4C, CHMP5, CHMP6, CHMP7, CFLAR, CYBB, CYLD, DNM1L, EIF2AK2, FADD, FAF1, FAS, FASLG, FTH1, FTL, GLUD1, GLUD2, GLUL, H2AB1, H2AB2, H2AB3, H2AC1, H2AC11, H2AC12, H2AC13, H2AC14, H2AC15, H2AC16, H2AC17, H2AC18, H2AC19, H2AC20, H2AC21, H2AC25, H2AC4, H2AC6, H2AC7, H2AC8, H2AJ, H2AX, H2AZ1, H2AZ2, HMGB1, HSP90AA1, HSP90AB1, IF1, IF10, IF13, IF14, IF16, IF17, IF2, IF21, IF4, IF5, IF6, IF7, IF8, IFNB1, IFNG, IFNGR1, IFNGR2, IFR1, IFR2, IL1A, IL1B, IL33, IRF9, JAK1, JAK2, JAK3, JMJD7-PLA2G4B, MACROH2A1, MACROH2A2, MAPK10, MAPK8, MAPK9, MLKL, NLRP3, PARP1, PGAM5, PLA2G4A, PLA2G4B, PLA2G4C, PLA2G4D, PLA2G4E, PLA2G4F, PPIA, PPID, PYGB, PYGL, PYGM, RBCK1, RIPK1, RIPK3, RNF103-CHMP3, RNF31, SHARPIN, SLC25A31, SLC25A4, SLC25A5, SLC25A6, SMPD1, SPATA2, SPATA2L, SQSTM1, STAT1, STAT2, STAT3, STAT4, STAT5A, STAT5B, STAT6, TLR3, TLR4, TNF, TNFAIP3, TNFRSF10A, TNFRSF10B, TNFRSF1A, TNFSF10, TRADD, TRAF2, TRAF5, TRPM7, TYK2, USP21, VDAC1, VDAC2, VDAC3, VPS4A, VPS4B, XIAP, ZBP1 |
| Pyroptosis | BAK1, BAX, CASP1, CASP3, CASP4, CASP5, CHMP2A, CHMP2B, CHMP3, CHMP4A, CHMP4B, CHMP4C, CHMP6, CHMP7, CYCS, ELANE, GSDMD, GSDME, GZMB, HMGB1, IL18, IL1A, IL1B, IRF1, IRF2, TP53, TP63 |

Supplementary Table S7. CMap analysis identified potential drugs for CRSwNP.

| Score | Name | Description |
| --- | --- | --- |
| -99.47 | itraconazole | Cytochrome P450 inhibitor |
| -99.58 | vinorelbine | Tubulin inhibitor |
| -99.65 | diethylstilbestro | Estrogen receptor agonist |
| -99.68 | atorvastatin | HMGCR inhibitor |
| -99.79 | deferiprone | Chelating agent |
| -99.82 | ZD-7288 | HCN channel blocker |
| -99.86 | ruxolitinib | JAK inhibitor |
| -99.89 | NS-3694 | Glutamate receptor antagonist |
| -99.93 | IKK-2-inhibitor | IKK inhibitor |
| -99.93 | PD-169316 | p38 MAPK inhibitor |
